# An Adipocyte MKK3–p38β Axis Preserves Insulin Responsiveness in Mice and Is Reciprocally Altered in Human Obesity

**DOI:** 10.1002/advs.202520388

**Published:** 2026-10-28

**Authors:** Edgar Bernardo, Nuria Matesanz, Jorge‐Luis Torres, Alba C. Arcones, Juan Ignacio Jiménez‐Loygorri, Alfonso Mora, Leticia Herrera‐Melle, Luis Leiva‐Vega, Maria Elena Rodriguez, Lourdes Hernández‐Cosido, Marta Leon, Paula Yong González, Teresa Durán, Tamara Parracho, Francisca Mulero, Rubén Nogueiras, J. Simon C. Arthur, Angel R. Nebreda, Roger J. Davis, Miguel Marcos, Guadalupe Sabio

**Affiliations:** ^1^ Centro Nacional de Investigaciones Oncológicas (CNIO) Madrid Spain; ^2^ Centro Nacional de Investigaciones Cardiovasculares Carlos III Madrid Spain; ^3^ Complejo Asistencial de Zamora Zamora Spain; ^4^ Bariatric Surgery Unit Department of General Surgery University Hospital of Salamanca Department of Surgery University of Salamanca Salamanca Spain; ^5^ Department of Physiology CiMUS University of Santiago de Compostela Santiago de Compostela Spain; ^6^ Division of Cell Signalling and Immunology Faculty of Life Sciences University of Dundee Dundee UK; ^7^ Institute for Research in Biomedicine (IRB Barcelona) The Barcelona Institute of Science and Technology Barcelona Spain; ^8^ Institució Catalana de Recerca i Estudis Avançats (ICREA) Barcelona Spain; ^9^ Program in Molecular Medicine University of Massachusetts Chan Medical School Massachusetts USA; ^10^ Department of Internal Medicine University Hospital of Salamanca‐IBSAL Salamanca Spain; ^11^ Department of Medicine University of Salamanca Salamanca Spain

**Keywords:** adipocytes, insulin resistance, MKK3/p38β signaling, obesity, type 2 diabetes mellitus

## Abstract

Obesity is a major risk factor for insulin resistance and type 2 diabetes. Adipocyte stress responses can either promote metabolic dysfunction or preserve insulin action, but the contribution of the p38 MAPK activator MKK3 to adipose tissue physiology remains poorly understood. Here, we show that adipose MKK3 expression increases early during high‐fat feeding and is also elevated in human obesity. In human visceral adipose tissue, MKK3 expression was positively associated with body mass index and leptin and inversely associated with HbA1c in unadjusted analyses. Using germline, Fabp4‐Cre, and Adipo‐Cre Mkk3‐deficient mice, together with p38β‐deficient mice and primary adipocytes, we found that loss of adipocyte MKK3 impairs insulin responsiveness. Adipo‐Cre;Mkk3^fl/fl^ (Adipo^Mkk3^) mice displayed reduced insulin‐stimulated glucose uptake selectively in subcutaneous white adipose tissue despite unchanged body composition and basal brown adipose tissue activity. Mechanistically, MKK3 or p38β deficiency increased p70S6K activity and inhibitory IRS1 Ser307 phosphorylation, reduced GLUT4 translocation and Akt activation, and these defects were partially rescued by rapamycin or the selective p70S6K inhibitor PF‐4708671. Together, these findings identify an adipocyte MKK3‐p38β‐p70S6K axis that helps preserve insulin signaling during nutritional stress.

## Graphical abstract

The adipocyte MKK3–p38β axis emerges as an adaptive stress‐response pathway during obesity. Human visceral adipose tissue analyses, adipocyte‐specific mouse models, and primary adipocytes show that the MKK3–p38β axis supports insulin responsiveness by restraining p70S6K‐dependent inhibitory IRS1 feedback and preserving AKT–GLUT4 signaling. This pathway defines a selective adipose mechanism that may help preserve glucose handling under nutritional stress.

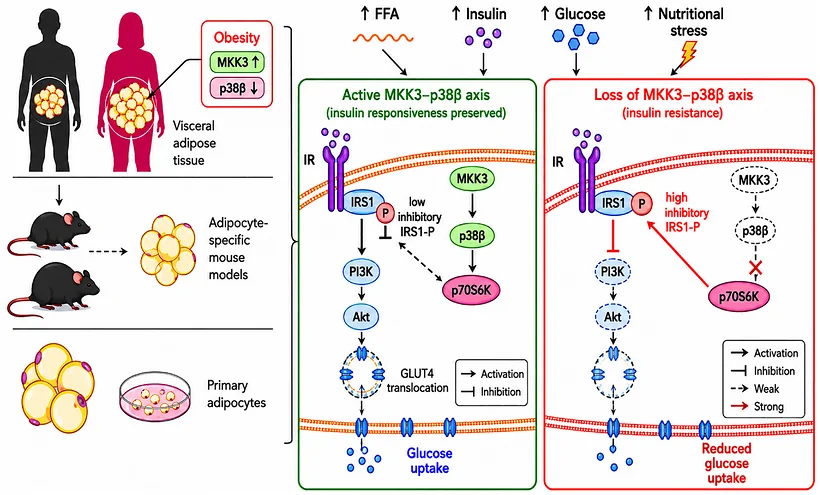

## 1 Introduction

Obesity, a major risk factor for insulin resistance and type 2 diabetes, is accompanied by chronic low‐grade inflammation in adipose tissue, immune‐cell infiltration, and increased production of inflammatory mediators [1]. Cytokines such as TNFα and IL‐6 impair insulin action in adipocytes [2, 3] and other insulin‐responsive tissues [4]. Stress‐activated protein kinases, including JNKs and p38 MAPKs, integrate these inflammatory and nutritional signals and can thereby influence both local adipose‐tissue function and systemic glucose homeostasis [4, 5, 6].

The p38 MAPK family comprises four family members encoded by different genes: p38α, p38β, p38γ and p38δ [7]. These kinases are activated predominantly through dual phosphorylation of their activation loop by the upstream MAP2Ks MKK3 and MKK6, although the relative contribution of each kinase depends on the members, tissue, and stimulus. These family members exert distinct, and in some cases opposing, metabolic functions. p38α has been implicated in insulin resistance in skeletal muscle and endothelial cells [8, 9], whereas myeloid p38α signaling controls inflammatory cytokine production and steatohepatitis [10], as well as hepatic inflammatory and metabolic remodeling [11, 12, 13]. p38γ and p38δ also regulate skeletal muscle mass and locomotor activity [14, 15], further illustrating the strong tissue and family member specificity of this pathway.

Adipose tissue provides a particularly clear example of this functional diversity. Previous work from our group showed that their upstream MKK6 regulates thyroid hormone‐induced browning of white adipose tissue [16], whereas p38α restrains brown‐adipose thermogenesis through inhibition of p38δ [7]. In addition, combined deletion of the p38 activators MKK3 and MKK6 in T cells promotes IL‐35‐dependent adipose browning and protects against diet‐induced obesity, demonstrating that p38 signaling can also regulate adipose adaptation through immune‐cell‐mediated mechanisms [17]. Beyond its roles in thermogenic remodeling and immune‐adipose communication, p38 signaling has also been implicated in adipocyte differentiation [18]. Early pharmacological studies also suggested that p38α/β might regulate glucose transport in adipocytes, although they produced apparently divergent effects depending on the inhibitor and experimental context [6, 19]. Subsequent genetic and pharmacological analyses questioned whether these effects reflected direct inhibition of p38: acute insulin‐stimulated glucose uptake was preserved in adipose tissue and skeletal muscle from p38β‐deficient mice, while the effect of the pharmacological inhibition of p38α/β SB203580 [20] on glucose transport was shown to vary independently of its inhibition of p38 activity [19]. Thus, these studies did not establish a clear member‐specific role for p38 in glucose transport and did not address whether p38β contributes to the maintenance of adipocyte insulin signaling during prolonged nutritional stress.

MKK3 and MKK6 are the principal upstream MAP2Ks that activate p38 MAPKs in adipose tissue. Their substrate preferences are not absolute, however, and depend on the stimulus, cellular environment, and relative abundance of the individual kinases [16, 21, 22]. In particular, p38β can be activated by both MKK3 and MKK6 [21, 23], and compensatory signaling between these MAP2Ks has been described in other tissues. In adipose tissue, MKK3 is substantially more abundant than MKK6, whereas MKK6 has been primarily associated with thermogenic remodeling of white adipose tissue [16]. Moreover, combined loss of MKK3 and MKK6 in myeloid cells disrupts macrophage–liver–adipose communication and the systemic control of thermogenesis [12]. Despite this evidence, whether MKK3 has an adipocyte‐autonomous role in metabolic adaptation and insulin signaling remains unknown.

Here, we investigated whether MKK3 contributes to adipocyte adaptation during obesity. We combined human visceral adipose tissue analyses with germline and adipocyte‐specific Mkk3‐deficient models, p38β‐deficient mice, and primary adipocytes. We show that loss of MKK3 in adipocytes impairs insulin‐stimulated glucose handling and that MKK3 and p38β restrain the p70S6K‐IRS1 inhibitory feedback pathway, which is a well‐established driver of adipose insulin resistance [24].

## 2 Results

### 2.1 Adipose MKK3 Expression Is Associated With Obesity‐related Metabolic Traits in Humans

Stress‐kinase pathways can have beneficial or detrimental effects in adipose tissue depending on the kinase, cell type, and metabolic context [25]. Whereas adipose p38α and MKK6 have established roles in thermogenic remodeling [7], the regulation and function of MKK3 in human obesity remain less defined [16]. To investigate whether the MKK3 pathway is altered in human obesity, we analyzed visceral adipose tissue and circulating metabolic markers in patients undergoing bariatric surgery and in a lower‐BMI surgical comparison group undergoing cholecystectomy. As expected, patients with obesity displayed higher circulating insulin, C‐peptide, and leptin levels and lower adiponectin levels than the cholecystectomy group (Figure 1A–D and Table S1). Adipose MKK3 expression correlated positively with BMI and with circulating insulin, C‐peptide and leptin, whereas it was inversely associated with HbA1c (Figure 1E–I) in unadjusted analysis. In a multivariable model adjusted for age, sex, BMI and T2D status, the inverse HbA1c association was attenuated and was not statistically significant (β = −0.20 HbA1c percentage points per doubling of MKK3; 95% CI, ‐0.56 to 0.16; p = 0.282). Insulin and C‐peptide represent closely related measures of insulin secretion; however, C‐peptide provides a more stable estimate of endogenous insulin production because it is not subjected to first‐pass hepatic extraction. Thus, the positive associations of MKK3 with both insulin and C‐peptide link increased adipose MKK3 expression to the compensatory hyperinsulinemia that accompanies insulin resistance. Together with its unadjusted inverse association with HbA1c, this pattern is compatible with the possibility that MKK3 is induced during obesity as part of an adaptive adipose response that may help preserve insulin responsiveness and glycaemic control under increasing metabolic stress. However, these associations do not establish a causal or mechanistic relationship.

**FIGURE 1:**
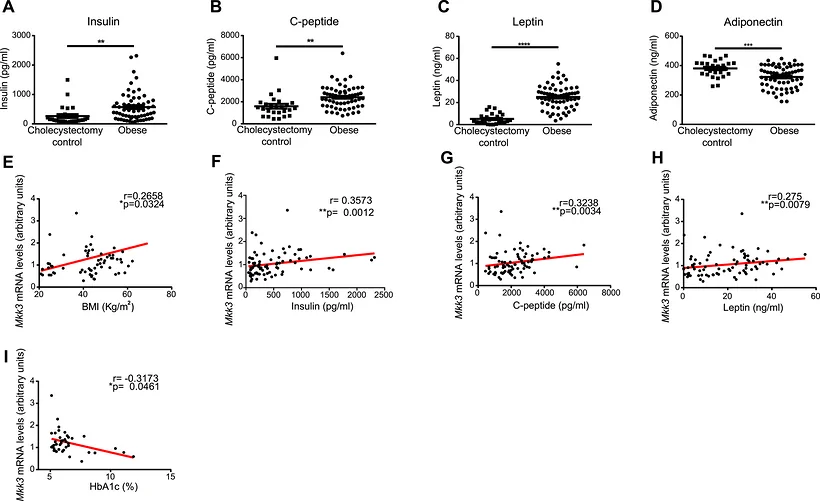
MKK3 expression in human adipose tissue is associated with obesity‐related traits. (A–D) Increased levels of insulin (A), C‐peptide (B), leptin (C), and reduced levels of adiponectin (D) in serum from cholecystectomy controls and bariatric surgery patients as measured by the Luminex test. Data are presented as mean ± SEM, n=18‐53, Student´s *t*‐test coupled with Welch´s correction, ^**^ *p* < 0.01, ^***^ *p* < 0.001, ^****^ *p* < 0.0001. E‐I. Mkk3 expression in visceral adipose tissue from cholecystectomy controls and bariatric‐surgery patients positively correlated with BMI (E) and circulating levels of insulin (F), C‐peptide (G), and leptin (H), and negatively correlated with HbA1c (I). Data are presented as discrete value comparisons between individuals and regression slope calculations, *n* = 48‐71, Spearman´s Rho, ^*^ *p* < 0.05, ^**^ *p* < 0.01.

Interestingly, analysis of GTEx data showed that MAP2K3 is expressed at substantially higher levels than MAP2K6 in human adipose tissue (Figure S1A,B), whereas MAP2K3, but not MAP2K6, expression correlated with several metabolic parameters (Figure S1C). In addition, human genetic data linked MAP2K3 gene variants to BMI, body weight, glycaemic traits and type 2 diabetes (Figure S1D and Table S2). Collectively, these complementary clinical, expression, and genetic observations identify MKK3 as an adipose stress‐response pathway associated with obesity‐related metabolic traits, although they do not establish a causal relationship in human obesity.

### 2.2 MKK3 Increased Early During High‐Fat Feeding and Its Loss Impairs Adipose Insulin Responsiveness

High‐fat diet induced *Mkk3* expression in epididymal WAT after only 3 weeks, and this increase was maintained at 8, 12, and 16 weeks (approximately 1.3‐, 1.7‐, 1.81‐, and 1.84‐fold, respectively; Figure S2A). This early increase supports the interpretation that MKK3 induction is an early adaptive response rather than simply a consequence of established obesity.

To investigate this potential adaptive response, we generated mice lacking *Mkk3*. Although total body weight was comparable between genotypes throughout the dietary challenge (Figure 2A), body‐composition analysis revealed a marked redistribution of tissue mass in *Mkk3*‐null mice, which displayed reduced fat mass together with increased lean mass and skeletal‐muscle weight, whereas liver weight remained unchanged (Figure S2D–I). Consistent with this broader systemic phenotype, germline Mkk3‐null mice also showed reduced energy expenditure and fatty‐acid oxidation across the light and dark phases, whereas food intake and locomotor activity were not significantly altered (Figure S2R–U). Despite their lower adiposity, fasting and fed glucose concentrations and glucose tolerance were comparable between genotypes (Figure 2B–D). In contrast, *Mkk3*‐null mice showed impaired glucose lowering during the ITT (Figure 2E), indicating reduced whole‐body insulin responsiveness.

**FIGURE 2:**
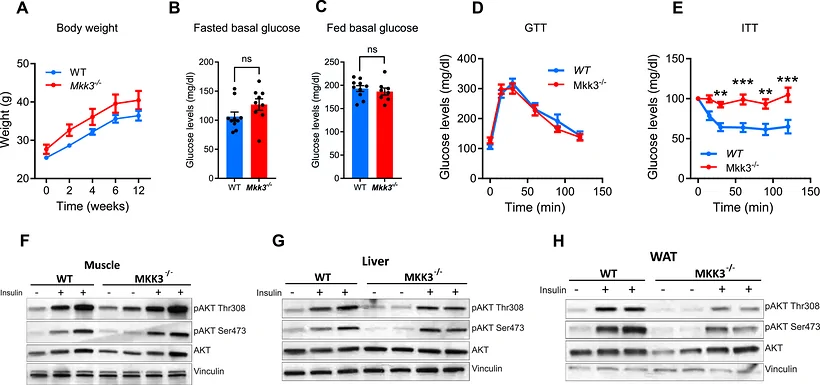
MKK3 deficient mice present insulin signaling impairment. (A) Body weight of 8‐week‐old WT and *Mkk3* ^−/−^ mice fed a HFD for 12 weeks. Data are presented as the mean ± SEM, *n* = 10, Two‐way ANOVA coupled with Bonferroni´s post‐test. (B, C) Overnight‐fasted (B) and fed (C) glycemic levels of WT and *Mkk3* ^−/−^ mice fed an HFD for 12 weeks showed no significant differences. Data are presented as mean ± SEM (*n* = 10), Student´s *t*‐test. (D) Glucose tolerance test (GTT) of HFD‐fed WT and *Mkk3* ^−/−^ mice. Data are presented as the mean ± SEM, n=10, Two‐way ANOVA coupled with Bonferroni´s post‐test. (E) Insulin tolerance test (ITT) showing that *Mkk3* ^−/−^ mice are insulin intolerant after intraperitoneal injection of insulin. Data are presented as mean ± SEM of the decrease with respect to initial glycemia, calculated as a percentage, *n* = 10, Two‐way ANOVA coupled to Bonferroni´s post‐test, ^**^ *p* < 0.01, ^***^ *p* < 0.001. (F–H) Immunoblots of AKT activation in muscle (F), liver (G), and white adipose tissue (WAT) (H). Fasted mice were treated with or without 1.5 IU/kg intraperitoneally injected insulin for 15 min.

To identify the tissue primarily contributing to this phenotype, we next examined insulin signaling in major insulin‐responsive organs. Insulin‐stimulated Akt phosphorylation was comparable in liver and skeletal muscle but was selectively reduced in WAT from *Mkk3*‐null mice (Figure 2F–H and Figure S2B). Thus, despite having lower fat mass, *Mkk3*‐null mice developed impaired systemic insulin responsiveness associated with a selective defect in adipose‐tissue insulin signalling.

### 2.3 Global MKK3 Deficiency Is Associated With a Pro‐Inflammatory Adipose Immune Profile

Macrophage accumulation is a characteristic feature of dysfunctional obese adipose tissue [26, 27]. Histological analysis revealed an increased number of crown‐like structures in WAT from HFD‐fed *Mkk3*‐null mice (Figure S2C). Consistent with this observation, flow cytometry analysis of the stromal vascular fraction showed increased macrophage infiltration and a higher M1‐like/M2‐like ratio (Figure S2P,Q), whereas expression of *Lyz2*, *Emr1*, *Ccl2*, *Ccl3*, *Il6* and *Tnfα* in bulk WAT was unchanged (Figure S2J–O). These data reveal a localized inflammatory phenotype that is not captured by bulk cytokine expression alone.

### 2.4 The Adipose Insulin‐Resistance Phenotype Is Not Reproduced by Myeloid MKK3 Deletion

To assess whether loss of MKK3 in hematopoietic cells was sufficient to impair insulin sensitivity, we generated bone‐marrow chimeras by transplanting WT or *Mkk3*‐null bone marrow into irradiated WT recipients and fed the mice with HFD. Donor reconstitution was confirmed by flow cytometry. Body and tissue weights, glucose tolerance, and insulin tolerance were comparable between groups (Figure S3A–H).

Consistent with the chimera experiments, Lyz2‐Cre‐mediated Mkk3 deletion did not alter body weight, adipose, liver, or muscle weights, or insulin responsiveness during HFD feeding (Figure S3I–N). These experiments indicate that deletion of MKK3 in the Lyz2‐expressing myeloid compartment is not sufficient to reproduce the metabolic phenotype, although the pro‐inflammatory myeloid bias observed in the germline knockout raises the possibility that MKK3 has additional functions in immune cells (Figure S2P,Q).

### 2.5 Adipocyte‐Specific Mkk3 Deficiency Reproduces Impaired Insulin Responsiveness

To determine whether the impaired insulin response of germline Mkk3‐null mice originated in adipose tissue, we first analysed Mkk3^Fabp4‐KO^ mice. MKK3 depletion was confirmed in WAT (Figure 3A). Under HFD feeding, mutant and control mice displayed comparable body weight, individual tissue weights, and glucose tolerance (Figure 3B,C and Figure S4A–D). Nevertheless, mice showed impaired glucose lowering during the ITT (Figure 3D). Consistent with the germline model, insulin‐stimulated Akt phosphorylation was preserved in liver and skeletal muscle but selectively reduced in WAT (Figure 3E–G and Figure S4T,U), indicating that loss of adipose MKK3 is sufficient to compromise systemic insulin responsiveness.

**FIGURE 3:**
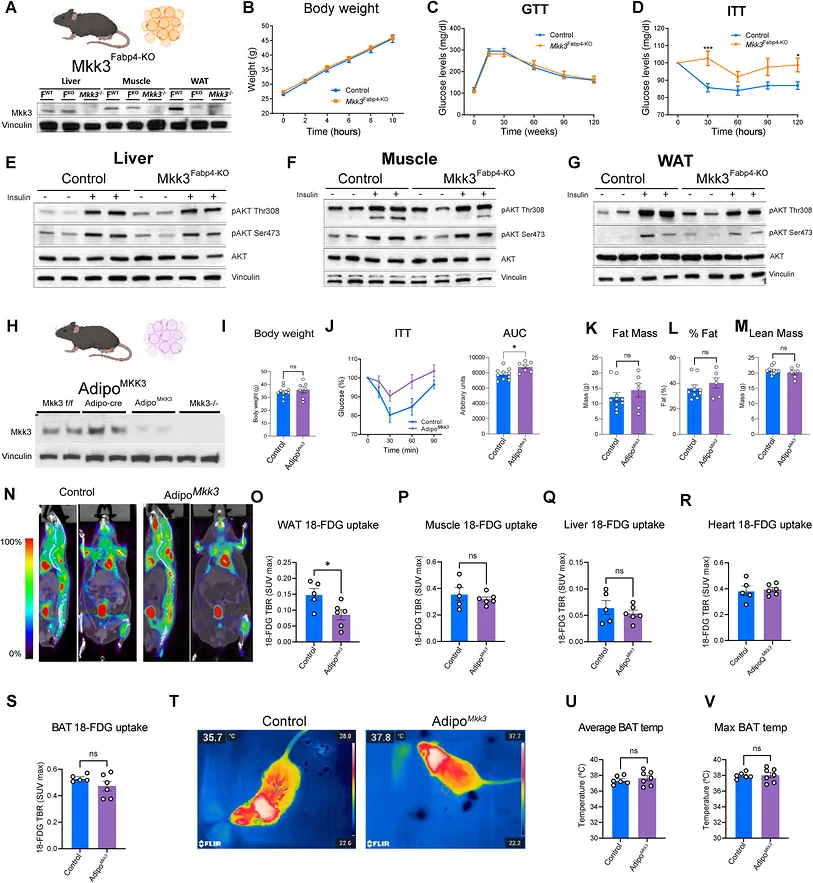
Adipocyte‐specific MKK3 deficiency impairs adipose insulin responsiveness. (A) Immunoblot showing MKK3 depletion in WAT from control (F^WT^) and Mkk3^Fabp4‐KO^ (F^KO^) mice. (B) Body weight of Fabp4‐Cre control and Mkk3^Fabp4‐KO^ mice during 10 weeks of HFD feeding. Data are presented as mean ± SEM, *n* = 8–11; two‐way ANOVA followed by Bonferroni's post hoc test. (C) GTT and (D) ITT in HFD‐fed Fabp4‐Cre control and Mkk3^Fabp4‐KO^ mice. Data are presented as mean ± SEM, *n* = 8–11; two‐way ANOVA followed by Bonferroni's post hoc test. ITT data are expressed as a percentage of basal glycemia. ^*^ *p* < 0.05, ^***^ *p* < 0.001. (E–G) Immunoblots showing insulin‐stimulated Akt activation in liver (E), skeletal muscle (F), and WAT (G) from HFD‐fed control and Mkk3^Fabp4‐KO^ mice. Fasted mice were treated with or without 1.5 U/kg insulin for 15 min before tissue collection. (H) Immunoblot confirming MKK3 depletion in eWAT from Adipo^*Mkk3*^ mice. I. Body weight after 10 weeks of HFD feeding. (J) ITT and corresponding AUC in HFD‐fed control and Adipo^*Mkk3*^ mice (*n* = 7–10). (K–M) Fat mass (K), percentage fat mass (L), and lean mass (M) measured by body‐composition analysis (*n* = 7–10). (N) Representative insulin‐stimulated ^18^F‐FDG PET‐CT image. (O–S) Quantification of ^18^F‐FDG uptake in scWAT (O), skeletal muscle (P), liver (Q), heart (R) and BAT (S), normalized to the blood pool and expressed as tissue‐to‐blood ratio (TBR) (*n* = 5–6). T. Representative infrared thermography images of interscapular BAT. U, V. Mean (U) and maximal (V) interscapular BAT surface temperature (*n* = 6–7).

To confirm this finding using an independent model targeting mature adipocytes, we generated Adipo*^Mkk3^* mice. Efficient MKK3 depletion was confirmed in WAT (Figure 3H). These mice reproduced the impaired insulin response observed in the Fabp4‐Cre model while maintaining body weight, fat mass, lean mass, individual tissue weights, and adipocyte histology comparable to those of littermate controls. Liver and muscle ectopic lipid content and macrophage infiltration were also unchanged (Figure 3I–M and Figure S4F‐M, R, S). A similar impairment in insulin responsiveness was also observed in female Adipo*^Mkk3^* mice after HFD feeding (Figure S4E). Together, these findings show that adipocyte‐specific MKK3 deficiency impairs insulin responsiveness in both sexes without altering body composition.

We next used insulin‐stimulated ^18^F‐FDG PET‐CT to identify the tissue responsible for this phenotype. Adipo*^Mkk3^* mice showed reduced glucose uptake specifically in WAT, whereas uptake in skeletal muscle, liver, heart, and BAT remained unchanged (Figure 3N–S). Consistent with the unchanged BAT glucose uptake, interscapular BAT temperature was also unaffected (Figure 3T–V). In contrast to the broader metabolic phenotype of germline *Mkk3*‐null mice, indirect calorimetry revealed no significant differences in energy expenditure, food intake, locomotor activity, or fatty‐acid oxidation between control and Adipo^Mkk3^ mice (Figure S4N–Q). Together, these findings demonstrate that MKK3 expression in mature adipocytes is required for normal adipose glucose handling and insulin responsiveness, independently of the changes in body composition and energy balance observed in germline *Mkk3*‐null mice.

### 2.6 MKK3 Deficiency Impairs Functional Insulin Responses in Primary Adipocytes

To determine whether the defect observed *in vivo* was adipocyte‐autonomous, we differentiated primary preadipocytes isolated from subcutaneous WAT of control and *Mkk3*‐null mice. Oil Red O staining and adiponectin expression were comparable between genotypes (Figure 4A–C), indicating that MKK3 deficiency did not impair adipocyte differentiation or lipid accumulation.

**FIGURE 4:**
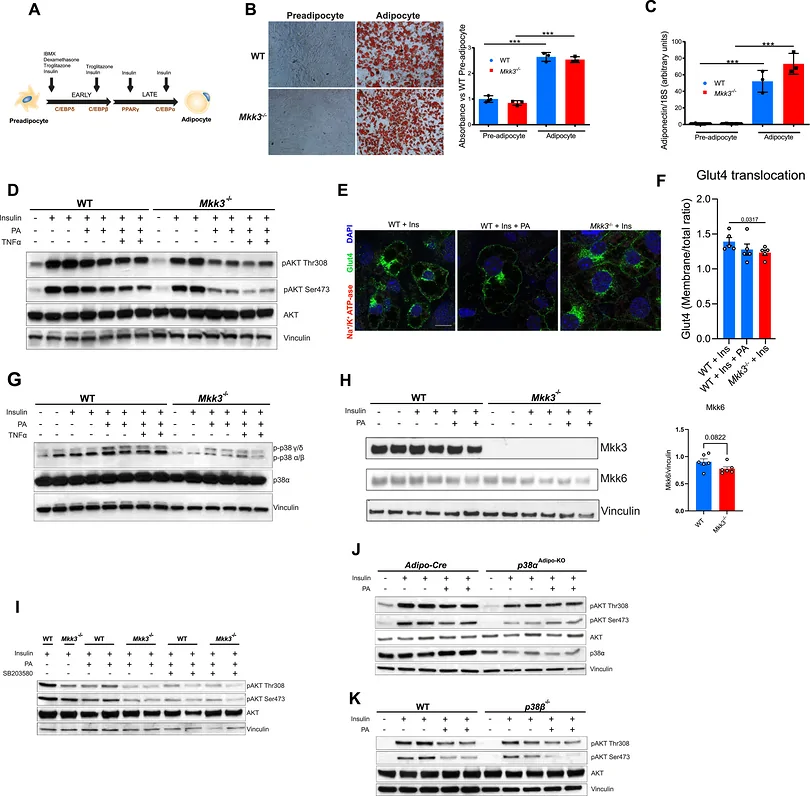
MKK3 deficiency impairs functional insulin responses in primary adipocytes. (A) Schematic represention of the mature primary adipocyte protocol of differentiation. (B) Representative images of Oil Red O staining (left) and quantification (right bar plot) of differentiated *Mkk3* ^−/−^ derived pre‐adipocytes showing no differences in lipid accumulation. The data are presented as colorimetric quantification. (C) Adipocyte‐specific Adiponectin expression was not altered in *Mkk3* ^−/−^ derived mature adipocytes. *n* = 3 replicates of 3 independent experiments, mean ± SEM, Student´s *t*‐test coupled with Bonferroni post‐test, ^***^ *p* < 0.001. (D) Western blot showing reduced activation of AKT in *Mkk3* ^−/−^ derived adipocytes treated with 0.5 mM palmitate and 10ng/mL TNFα. (E) Representative immunofluorescence of WT and *Mkk3* ^−/−^ adipocytes stimulated with insulin or insulin and 0.5mM palmitate. Staining Na+/K+ ATP‐ase (red), GLUT4 (green), and DAPI (blue). Scale bar indicates 10µm. (F) Quantification of GLUT4 translocation vs cytoplasmatic GLUT4, n=5 adipocytes across 3 different replicates. (G) Immunoblot showing reduced phosphorylation of p38α/β in *Mkk3* ^−/−^ derived adipocytes after treatment with 0.5mM of palmitate 10ng/mL TNFα. (H) Immunoblot showing unchanged levels of MKK6 in *Mkk3* ^−/−^ derived adipocytes after treatment with 0.5 mM palmitate. Right bar plot indicates MKK6 quantification (I) Western blot showing that *Mkk3* ^−/−^ adipocytes AKT activation is not further reduced when treated for 24 h with 10µM p38α/β specific inhibitor SB203580, palmitate 0.5mM. (J) Immunoblot showing reduced AKT phosphorylation in mature adipocytes derived from white adipose tissue‐specific p38α knockout mice after 0.5mM palmitate treatment for 24 h. (K) Western blot showing reduced AKT activation in mature p38β^−/−^ mice‐derived adipocytes after 24 h of 0.5 mM palmitate treatment. (D–K) Adipocytes starved for 2 h were stimulated or not with 10 nM insulin for 10 min.

Despite normal differentiation, *Mkk3*‐null adipocytes showed reduced insulin‐stimulated Akt phosphorylation, and this defect was further exacerbated by palmitate, either alone or in combination with TNFα (Figure 4D). The impaired signaling response was accompanied by reduced insulin‐induced GLUT4 translocation to the plasma membrane (Figure 4E,F). Notably, the magnitude of the GLUT4 defect in untreated Mkk3‐null adipocytes was comparable to that induced by palmitate in control cells. These findings demonstrate that MKK3 deficiency directly compromises insulin action in adipocytes and increases their susceptibility to obesogenic stress.

### 2.7 MKK3 and p38β Participate in a Common Adipocyte Insulin‐Signaling Pathway

Having established that MKK3 is required for insulin responsiveness in adipocytes, we next investigated which p38 MAPK family members might mediate this effect. MKK3 deficiency reduced the phospho‐p38 signal corresponding predominantly to p38α and p38β under basal and insulin‐stimulated conditions, and this reduction was particularly evident following exposure to palmitate or palmitate plus TNFα, whereas no compensatory increase in MKK6 protein abundance was observed in MKK3‐deficient adipocytes (Figure 4G,H).

Pharmacological inhibition of p38α/β with SB203580 reduced insulin‐stimulated Akt phosphorylation in WT adipocytes but did not further exacerbate the defect observed in *Mkk3*‐null cells, suggesting that MKK3 deficiency and p38α/β inhibition converge on a common insulin‐signaling pathway (Figure 4I). We therefore examined insulin responsiveness in adipocytes lacking either p38α or p38β. Adipocytes deficient in p38α displayed reduced insulin‐stimulated Akt phosphorylation, whereas p38β‐null adipocytes showed impaired Akt activation that was further exacerbated by palmitate treatment (Figure 4J,K). Thus, although MKK3 may regulate more than one p38 isoform, the phenotype of p38β‐deficient adipocytes closely reproduced the impaired insulin response observed after MKK3 loss.

We next examined the relevance of p38β in human adipose tissue. GTEx analysis showed that *MAPK11*, encoding p38β, is one of the most highly expressed p38 family members in adipose tissue (Figure S5A–D). In our cohort, visceral adipose *MAPK11* expression was inversely associated with BMI, circulating C‐peptide and leptin levels, and adipose MKK3 expression (Figure 5A–D). Thus, whereas adipose MKK3 increased with obesity‐related metabolic traits, *MAPK11* showed the opposite pattern. Human genetic associations linking MAPK11 to BMI and T2D provided additional support for examining p38β (Figure S5E,F and Table S3).

**FIGURE 5:**
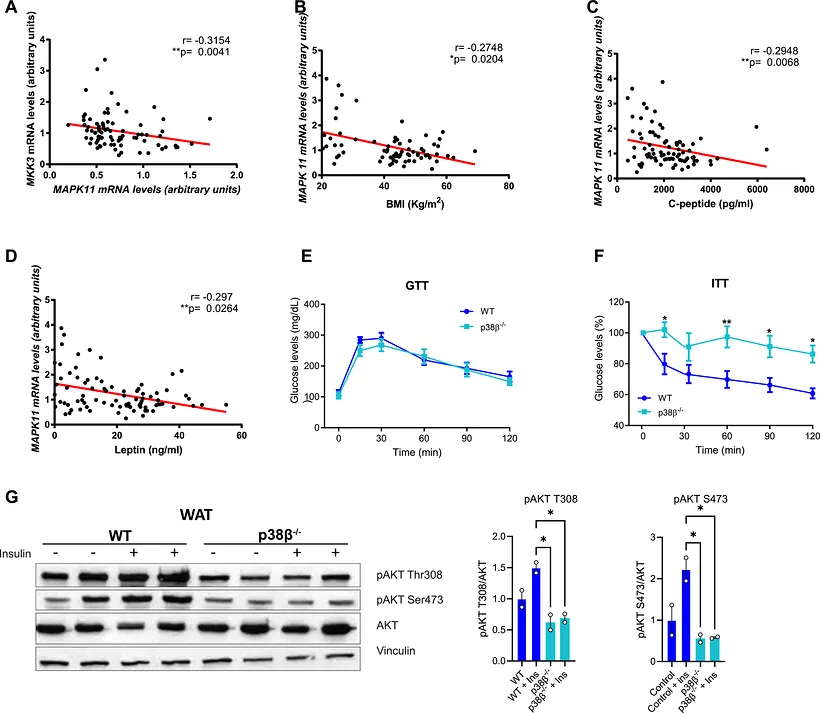
p38β expression is reciprocally associated with MKK3 and obesity‐related metabolic traits and is required for normal insulin responsiveness. (A–D) MAPK11 (p38β) expression in human visceral adipose tissue is inversely associated with MKK3 expression (A), BMI (B), circulating C‐peptide (C), and leptin (D). Data are shown for individual participants from the lower‐BMI cholecystectomy comparison group and the bariatric‐surgery group; *n* = 71, Spearman's rho, ^*^ *p* < 0.05, ^**^ *p* < 0.01. E. GTT and F. ITT in HFD‐fed WT and p38β^−^^/^^−^ mice. (G) Representative immunoblot (left) and quantification (right) of basal and insulin‐stimulated Akt phosphorylation in WAT from HFD‐fed WT and p38β^−^^/^^−^ mice. Fasted mice were stimulated with insulin before tissue collection. Data are presented as mean ± SEM; ^*^ *p* < 0.05.

To determine whether p38β was functionally required for insulin responsiveness in vivo, WT and p38β‐null mice were fed a HFD. HFD‐fed p38β‐null mice displayed normal glucose tolerance but impaired glucose lowering during the ITT (Figure 5E,F). Consistent with this systemic phenotype, basal and insulin‐stimulated Akt phosphorylation was reduced in WAT (Figure 5G), reproducing key features of the Mkk3‐deficient models. Histological analysis further revealed increased leukocyte infiltration and smaller adipocytes in WAT from p38β‐null mice, whereas liver and BAT morphology remained unchanged (Figure S5G).

To determine whether loss of p38β in hematopoietic cells contributed to this phenotype, we generated bone‐marrow chimeras by transplanting WT recipients with either WT or p38β‐null bone marrow (Figure S5H). Mice receiving p38β‐null hematopoietic cells showed no differences in body weight or insulin responsiveness compared with controls (Figure S5I,J). Thus, although global p38β deficiency is associated with an altered immune landscape in adipose tissue, loss of p38β in hematopoietic cells alone is not sufficient to reproduce the metabolic phenotype, supporting a predominant contribution from non‐hematopoietic cells.

### 2.8 MKK3‐p38β Signaling Restrains the p70S6K‐IRS1 Inhibitory Feedback Pathway

Serine phosphorylation of IRS1 constitutes an important negative‐feedback mechanism in insulin signaling [28]. p70S6K promotes inhibitory serine phosphorylation of IRS1, thereby attenuating downstream insulin signaling. Compared with control cells, *Mkk3*‐null adipocytes displayed increased IRS1 Ser307 phosphorylation under both basal and palmitate‐treated conditions (Figure 6A). This was accompanied by increased phosphorylation of p70S6K and its downstream substrate S6 (Figure 6B), suggesting that enhanced p70S6K activity contributes to the increased inhibitory phosphorylation of IRS1. A similar increase in p70S6K and S6 phosphorylation was observed in p38β‐null adipocytes (Figure 6C), indicating that MKK3 and p38β deficiency converge on the same downstream signaling pathway.

**FIGURE 6:**
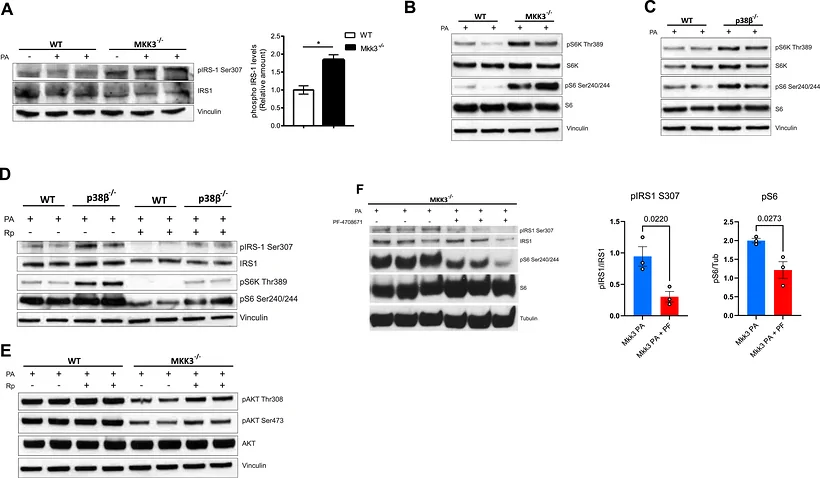
MKK3–p38β signaling restrains the p70S6K–IRS1 inhibitory feedback pathway. (A) Immunoprecipitation/immunoblot analysis (left) and quantification (right) showing increased IRS1 Ser307 phosphorylation in Mkk3^−^^/^^−^ adipocytes under basal conditions and following 0.5 mM palmitate treatment. Data are presented as mean ± SEM, *n* = 3; Student's *t*‐test with Bonferroni correction, ^*^ *p* < 0.05. (B, C) Immunoblots showing increased phosphorylation of p70S6K and its downstream target S6 in adipocytes derived from Mkk3^−^^/^^−^ (B) and p38β^−^^/^^−^ (C) mice following treatment with 0.5 mM palmitate. (D) Immunoblot analysis showing increased IRS1 Ser307, p70S6K, and S6 phosphorylation in p38β^−^^/^^−^ adipocytes and partial reversal following treatment with 10 µM rapamycin. (E) Immunoblot showing partial restoration of insulin‐stimulated Akt phosphorylation in Mkk3^−^^/^^−^ adipocytes treated with 10 µM rapamycin. (F) Representative immunoblot and quantification showing reduced IRS1 Ser307 and S6 phosphorylation in palmitate‐treated Mkk3^−^^/^^−^ adipocytes following treatment with the selective p70S6K inhibitor PF‐4708671.

We next tested whether suppressing this pathway could restore insulin responsiveness. Rapamycin reduced p70S6K activity and IRS1 Ser307 phosphorylation in p38β‐null adipocytes and partially restored insulin‐stimulated Akt phosphorylation in *Mkk3*‐null cells (Figure 6D,E). Because rapamycin broadly inhibits mTORC1 signaling, we then used the selective p70S6K inhibitor PF‐4708671 [29]. PF‐4708671 reduced both S6 phosphorylation and IRS1 Ser307 phosphorylation in *Mkk3*‐null adipocytes (Figure 6F). Together, these findings support a model in which MKK3–p38β signaling preserves insulin responsiveness by restraining p70S6K‐dependent inhibitory feedback on IRS1.

## 3 Discussion

Our study identifies adipocyte MKK3 as an adaptive component of insulin signaling during nutritional stress. Germline Mkk3 deficiency and two independent adipocyte‐targeted models consistently showed impaired insulin responsiveness. Importantly, Adipo*^Mkk3^* mice reproduced the ITT phenotype without changes in body composition, individual tissue weights, BAT activity, or whole‐body energy balance. Insulin‐stimulated ^18^F‐FDG PET‐CT localized the functional defect to reduced glucose uptake in subcutaneous WAT, while uptake in skeletal muscle, liver, heart, and BAT remained unchanged. Together with the impaired Akt activation and GLUT4 translocation observed in primary Mkk3‐null adipocytes, these findings demonstrate that loss of MKK3 in mature adipocytes is sufficient to compromise adipose insulin action. A similar impairment was observed in female Adipo*^Mkk3^* mice after prolonged HFD feeding, indicating that this phenotype is present in both sexes.

Mechanistically, our data place the p70S6K–IRS1 inhibitory feedback pathway downstream of MKK3 signaling. *Mkk3*‐null adipocytes showed increased p70S6K activity, enhanced S6 phosphorylation, and increased IRS1 Ser307 phosphorylation. Pharmacological inhibition of mTORC1 with rapamycin reduced p70S6K signaling and IRS1 phosphorylation and partially restored insulin‐stimulated Akt activation. More selective inhibition of S6K1 with PF‐4708671 also reduced S6 phosphorylation and IRS1 Ser307 phosphorylation, supporting a contribution of excessive S6K1 activity to the inhibitory feedback generated by MKK3 deficiency. Although IRS1 Ser307 phosphorylation is regulated by mTORC1–S6K1 signaling and has been linked to insulin resistance, other IRS1 residues, including Ser1101, are better‐established direct S6K1 targets, and the kinase responsible for Ser307 phosphorylation can be context dependent. Our data therefore establish that Ser307 phosphorylation is S6K1‐dependent under these conditions but do not demonstrate that S6K1 directly phosphorylates this residue in adipocytes. This is consistent with the established role of sustained mTORC1–S6K1 signaling in attenuating IRS‐dependent insulin signaling. Constitutive activation of the TSC–Rheb–mTORC1–S6K pathway following loss of TSC1 or TSC2 induces negative feedback on IRS1/2 and markedly reduces PI3K–Akt activation, whereas S6K1 deficiency protects against diet‐induced insulin resistance and reduces IRS1 Ser307 phosphorylation [30, 31, 32].

Our genetic data further identify p38β as a functionally relevant component of this pathway. Loss of either MKK3 or p38β impaired insulin‐stimulated Akt activation and increased p70S6K signaling in adipocytes, while p38β‐null mice reproduced key features of the *Mkk3*‐deficient metabolic phenotype. How p38β restrains mTORC1–S6K1 signaling in adipocytes remains to be determined. However, a previously described p38β–PRAK–Rheb pathway provides a particularly plausible mechanistic link. During energetic stress, p38β activates PRAK/MK5, which directly phosphorylates Rheb at Ser130, impairing its nucleotide‐binding capacity and thereby limiting Rheb‐dependent mTORC1 activation and S6K1 phosphorylation. Accordingly, loss of either p38β or PRAK prevents the normal suppression of mTORC1/S6K1 during energy depletion. Although this mechanism has not been tested in adipocytes, it is strikingly consistent with the increased S6K1 activity observed here following MKK3 or p38β deficiency and raises the possibility that a similar p38β–PRAK–Rheb module restrains mTORC1 during nutritional or lipotoxic stress in adipocytes [33].

Importantly, p38‐dependent regulation of mTOR is highly context‐dependent. Under arsenite‐induced stress, p38β has been shown to directly phosphorylate Raptor at Ser771 and Ser863 and thereby enhance, rather than suppress, mTORC1 activity, demonstrating that the same p38 family member can exert opposing effects on mTORC1 depending on the stimulus and available substrates. Other p38 family members can also directly engage the mTOR machinery through distinct mechanisms. We previously showed that p38γ and p38δ phosphorylate the endogenous mTOR inhibitor DEPTOR in cardiomyocytes, promoting its degradation and consequent mTOR activation. Together, these observations suggest that the functional coupling between p38 MAPKs and mTORC1 is determined by the specific p38 family member, cellular context, and upstream stress signal [34, 35].

Additional mechanisms upstream of Akt cannot be excluded. p38 family members have been shown to regulate PI3K signaling independently of mTORC1. For example, p38δ directly inhibits PKD1 in pancreatic β cells, whereas in neutrophils the p38δ–PKD1 pathway regulates PTEN activity through phosphorylation of the PI3K regulatory subunit p85α. Although these mechanisms were identified in different cell types and should not be directly extrapolated to adipocytes, they illustrate how individual p38 family members can modulate insulin‐ and PI3K‐related signaling at multiple levels. Thus, MKK3–p38β may influence adipocyte insulin responsiveness through additional targets beyond the mTORC1–S6K1 axis [36, 37].

Together, our results support a model in which MKK3–p38β signaling limits excessive p70S6K‐dependent negative feedback during nutritional stress, thereby preserving adipocyte insulin responsiveness. However, the molecular substrate linking p38β to mTORC1–S6K1 in adipocytes remains to be identified, and our data do not imply that all consequences of MKK3 deficiency are mediated exclusively through p38β or S6K1.

The contrast between MKK3 and MKK6 in adipose tissue further illustrates this context dependence. MKK6 deficiency promotes white‐adipose browning and protects against diet‐induced obesity [16], whereas adipocyte MKK3 deficiency did not affect thermogenesis, energy expenditure, or body composition but selectively impaired insulin responsiveness. Thus, although MKK3 and MKK6 can activate overlapping p38 family members, they appear to control distinct aspects of adipocyte adaptation to nutritional stress. Moreover, compensatory activation of p38γ and p38δ following MKK6 loss has been demonstrated in other tissues [16, 21, 22], indicating that MKK3 and MKK6 are not simply redundant upstream kinases. Such compensatory rewiring may help explain why these MAP2Ks can exert divergent, and in some contexts opposing, biological effects.

Previous studies did not establish a clear member‐specific role for p38 in adipocyte glucose transport. Pharmacological inhibition of p38 was reported to modify glucose uptake in insulin‐resistant adipocytes [6], but subsequent pharmacological and genetic analyses indicated that some effects of SB203580 on glucose transport occurred independently of p38 inhibition and that acute insulin‐stimulated glucose uptake was preserved in adipose tissue and skeletal muscle from p38β‐null mice [19]. Our data demonstrate a role for p38β in maintaining adipocyte insulin signaling during nutritional stress rather than in determining acute glucose transport under basal conditions.

The comparison between germline and adipocyte‐specific Mkk3‐deficient mice also reveals that systemic MKK3 loss produces additional phenotypes that are mechanistically distinct from the adipocyte insulin‐signaling defect. Reduced fat mass, increased lean and skeletal‐muscle mass, and a more pro‐inflammatory adipose immune profile were observed in germline Mkk3‐null mice but not in mice lacking MKK3 in adipose tissue. By contrast, impaired adipose insulin signaling was shared by all three Mkk3‐deficient models. This separation indicates that the insulin‐resistance phenotype is adipocyte autonomous, as was confirmed by the in vitro studies, whereas the changes in body composition and adipose inflammation probably arise from functions of MKK3 in other tissues or from altered inter‐organ communication.

Global MKK3 deficiency increased macrophage accumulation and shifted the adipose macrophage compartment toward a more pro‐inflammatory profile. However, this inflammatory phenotype was not reproduced by adipocyte‐specific Mkk3 deletion and was not sufficient to explain the insulin‐signaling defect. Moreover, neither Lyz2‐Cre‐mediated Mkk3 deletion nor transplantation of *Mkk3*‐null bone marrow reproduced the impaired insulin response. These negative results do not imply that MKK3 is irrelevant in immunity. Germline Mkk3 deficiency impairs IL‐12 production by macrophages and dendritic cells [38], MKK3 activates p38α in LPS‐stimulated neutrophils to regulate adhesion and TNF production [39], and MKK3 loss modifies macrophage mitochondrial function and inflammatory output [40]. Our previous studies also showed that combined MKK3/MKK6 deficiency in myeloid cells disrupts the macrophage‐liver‐BAT IL‐12‐FGF21 axis [12], whereas the MKK3/6‐p38γ/δ pathway regulates macrophage TNF translation during endotoxin‐induced hepatitis [41]. Combined MKK3/MKK6 deletion in T cells also modifies adipose inflammation and thermogenesis through IL‐35 [17]. MKK6 compensation, incomplete targeting of relevant immune populations, or the requirement for coordinated effects in several lineages may therefore explain why isolated loss of MKK3 in Lyz2‐expressing cells did not reproduce the phenotype of the germline knockout.

MKK3 functions in skeletal muscle and liver may similarly contribute to features that were unique to the germline model. The MKK3‐p38α pathway promotes terminal myogenic differentiation in C2C12 cells [42], and our genetic studies demonstrate that p38 family members regulate skeletal‐muscle mass, strength, and locomotor activity through PGC1α‐ and IL‐15‐dependent mechanisms [14, 15]. The absence of increased lean and skeletal‐muscle mass in both adipocyte‐specific models therefore raises the possibility that altered MKK3‐p38 signaling in muscle contributes to the body‐composition phenotype of germline Mkk3‐null mice. In hepatocytes, MKK3‐dependent p38α activation regulates cAMP‐induced MRP2 trafficking, with compensatory MKK6 upregulation after MKK3 loss [43]. At the organismal level, p38γ/δ‐dependent neutrophil recruitment regulates hepatic inflammation and circadian metabolism [11, 12, 13], while myeloid p38 signaling sustains liver‐adipose communication [12]. Tissue‐specific Mkk3 models will be required to determine the relative contribution of these pathways to the systemic phenotype.

The observations in human adipose tissue provide a clinically relevant context for our experimental findings. Adipose MKK3 expression increased with BMI and correlated positively with leptin and C‐peptide, linking MKK3 induction to the compensatory hyperinsulinemia that accompanies insulin resistance. MKK3 expression was also inversely associated with HbA1c in the unadjusted analysis, but this association was attenuated and was not statistically significant after multivariable adjustment. Moreover, *MAPK11*/p38β expression decreased with increasing obesity. These reciprocal changes are consistent with the possibility that increased MKK3 represents an adaptive response that helps preserve signaling through a reduced p38β pool during metabolic stress. The association of *MAP2K3*/MKK3 with BMI is also consistent with previous human genetic and adipose‐expression data [44].

Several limitations should be considered. The human cohort was cross‐sectional and therefore cannot establish the direction or causality of the observed associations. The cholecystectomy group represented a lower‐BMI surgical comparison group rather than a uniformly lean and metabolically healthy control population. Moreover, the inverse association between MKK3 and HbA1c was attenuated after adjustment for age, sex, BMI, type 2 diabetes status, and antidiabetic treatment; however, residual confounding related to clinical history, duration of obesity, and medication type cannot be excluded. At the mechanistic level, although the parallel phenotypes of MKK3‐ and p38β‐deficient models support their participation in a common pathway, direct biochemical evidence that p38β mediates all downstream effects of MKK3 is not provided. Contributions from p38α or other p38 family members therefore remain possible. Nevertheless, the consistency of the results across mouse models, primary adipocytes, and human adipose tissue supports the biological relevance of the pathway.

Overall, our results support a model in which early induction of adipocyte MKK3 during obesity helps preserve insulin responsiveness by engaging p38β and restraining p70S6K‐dependent inhibitory phosphorylation of IRS1. They also illustrate why systemic manipulation of p38 signaling can produce complex or apparently contradictory metabolic effects: individual MAP2Ks and p38 family members perform distinct functions in different cell types and metabolic settings. Rather than broad inhibition of p38α/β, selective modulation of the adipocyte MKK3‐p38β‐p70S6K pathway may provide a more directed strategy for understanding and potentially improving adipose insulin sensitivity, although its therapeutic relevance will require further investigation.

## 4 Materials and Methods

### 4.1 Human Studies

The study included adults with BMI ≥ 35 kg/m^2^ undergoing elective bariatric surgery at the University Hospital of Salamanca and a lower‐BMI surgical comparison group undergoing laparoscopic cholecystectomy for gallstone disease. The cholecystectomy group was not considered a uniformly healthy or lean population control. Exclusion criteria included alcohol use disorder or excessive alcohol intake (> 30 g/day in men and > 20 g/day in women) and chronic hepatitis B or C. The study was approved by the Ethics Committee of the University Hospital of Salamanca, and all participants provided written informed consent. Fasting venous blood was collected before surgery, and visceral adipose tissue was obtained under direct vision during the same surgical procedure. No postoperative samples were included. Demographic, clinical, medication, and metabolic characteristics are reported in Table S1. Only visceral adipose tissue was available for gene‐expression analyses.

Data were collected on demographic information (age and sex), anthropometric measurements (BMI), smoking and alcohol history, coexisting medical conditions, and medication use. Before surgery, fasting venous blood samples were collected for measuring complete cell blood count, total bilirubin, aspartate aminotransferase (AST), alanine aminotransferase (ALT), total cholesterol, high‐density lipoprotein (HDL), low‐density lipoprotein (LDL), triglycerides, alkaline phosphatase (AP), and gamma‐glutamyl transpeptidase (GGT).

### 4.2 Animal Models

MKK3 deficient mice (*Mkk3* ^−/−^, B6.129‐*Map*2*k*3^1Flv^) generation was previously described [38], and kindly donated by Dr. Roger J Davis from the University of Massachusetts. Animals were maintained on a C57BL/6J background (back‐crossed 10 generations), and genotype was confirmed by PCR analysis of genomic DNA.

To generate tissue‐specific knockout mice, we used the *Cre‐LoxP* system. The *Cre* recombinase can be expressed in a tissue‐specific manner using a tissue‐specific promoter such as *Fabp4, Adipo* (for adipose tissue), *and Lyz2* (for myeloid cells).

Mice lacking MKK3 specifically in adipose tissue were generated as follows. Mice with a germ‐line mutation in the *Map2k3* gene and LoxP elements inserted into two introns (Map2k3LoxP) were generated after homologous recombination in ES cells. ES cells were electroporated with a vector and selected with 200 µg/mL G418 and 2 µM gancyclovir. Several correctly targeted ES cell clones were identified by Southern blot and PCR. These ES cell clones were injected into C57BL/6J blastocysts to create chimeric mice that transmitted the mutated *Map2k3* allele through the germ line. The Flp NeoR cassette was excised by crossing these mice with ACTB:FLPe B6;SJL mice, which express a FLP1 recombinase gene under the direction of the human ACTB promoter. These animals were crossed with mice expressing *Cre* recombinase under the control of the adipose tissue‐specific *Fabp4* promoter (*Fabp4‐Cre*) [45] mice on the C57BL/6J background to generate mice lacking MKK3 in adipose tissue. To reduce the number of *LoxP* sequence recombinations needed and achieve a better depletion of *Mkk3* gene, we crossed these mice with full knockout *Mkk3* mice (*Mkk3* ^−^ *^/^* ^−^), obtaining *Fabp4‐Cre^+^Mkk3^f/^* ^−^ mice (F^KO^ mice). Littermates without the conditional *Mkk3* allele (*Fabp4‐Cre^+^Mkk3^+/^* ^−^) were used as control mice (F^WT^). Genotype was confirmed by PCR analysis of genomic DNA. Alternatively, mice lacking MKK3 specifically in adipose tissue were generated by crossing *Mkk3^f/f^* mice with mice expressing *Cre* recombinase under the control of the adipose tissue‐specific *Adiponectin* promoter (*Adipo‐Cre*) [46]. *Adipo‐Cre* mice were crossed with Mkk3 floxed mice (Adipo*^Mkk3^*), and littermates without the conditional *Mkk3* floxed allele were used as controls. Genotype was confirmed by PCR analysis of genomic DNA. Mice lacking MKK3 specifically in the myeloid lineage tissue were generated by crossing *Mkk3^f/f^* mice with mice expressing *Cre* recombinase under the control of the myeloid lineage‐specific *Lyz2* promoter (*Lyz2‐Cre*), previously described [47]. These mice were also crossed with full knockout *Mkk3* mice (*Mkk3* ^−^ *^/^* ^−^), obtaining *Lyz2‐Cre^+^Mkk3^f/^* ^−^ mice (Lyz^KO^ mice). Littermates without the conditional *Mkk3* allele (*Lyz2‐Cre^+^Mkk3^+/^* ^−^) were used as control mice (Lyz^WT^). Genotype was confirmed by PCR analysis of genomic DNA. Mice lacking p38β in the whole body were kindly donated by Dr. Angel Nebreda from IRB Barcelona. They were originally generated as p38α^f/f^ p38β^−/−^ and p38α^f/f^ p38β^+/+^ were used as controls.

All mice were housed in a specific pathogen‐free (SPF) or quarantine animal facility on a 12 h light/dark cycle at constant temperature and humidity at Centro Nacional de Investigaciones Oncológicas (CNIO), Centro Nacional de Investigaciones Cardiovasculares (CNIC), Universidad de Santiago de Compostela and Institut de Recerca de Barcelona (IRB) supervised by veterinarian staff. All mice were maintained on a C57BL/6J background (back‐crossed 10 generations). All animal experiments conformed to EU Directive 2010/63EU and Recommendation 2007/526/EC, enforced in Spanish law under Real Decreto 53/2013.

Radiation chimeras using congenic C57BL/6J, *Mkk3* ^−/−^ or p38β^−/−^ (CD45.2) and B6.SJL (CD45.1) mice were generated by exposing 8‐week‐old B6.SJL recipient mice to two doses of ionizing radiation (6.5 Gy) and reconstituting with 2 × 10^7^ cells from either WT, *Mkk3* ^−/−^ or p38β^−/−^ donor BM by tail vein injection. After BM injection, mice were treated with antibiotics in the drinking water ad libitum, 1.5 mg/mL gentamicin (Sigma), 1.5 mg/mL kanamycin (Sigma), and 1.5 mg/mL ampicillin (Sigma) for two months.

All animals were weighed, fasted for 16 h, bled from the cheek, and stimulated without/with 1.5 U/kg of insulin injected i.p 10 min before sacrifice. Tissues were weighed and collected, and the tibia was measured using a digital caliper (Ratio 6369 HS) to normalize organ weight to tibial length. For protein extraction, tissues were snap‐frozen in liquid nitrogen (LN2), for RNA isolation, samples were submerged in RNAse inhibitor solution (RNAseZAP, Thermo Fisher Scientific) prior to LN2 snap‐freeze, and for histology evaluation, tissues were fixed in 10% formalin solution or included in O.C.T (TissueTek) and snap‐frozen in LN2.

### 4.3 Study Reconstitution Efficiency by Flow Cytometry

To check implementation of donor BM and proper depletion of recipient BM in radiation chimeras experiments, stromal vascular fraction (SVF) was isolated after type‐A collagenase (Roche) disaggregation for 45 min at 37°C on an end‐to‐end water bath. Cellular stromal precipitation was stained at a 1:200 dilution with anti‐CD45.2 and CD45.1 conjugated with PerCP‐5.5 and Pacific Blue fluorophores (BD Biosciences), respectively, and analyzed by flow cytometry.

For adipose immune profiling, the stromal vascular fraction was isolated from epididymal WAT by collagenase digestion. CD45+ myeloid cells and F4/80+CD11b+ macrophages were quantified by flow cytometry, and the relative pro‐inflammatory and anti‐inflammatory macrophage profiles were assessed using CD11c and CD206, respectively. Results were expressed as macrophage abundance and the M1‐like/M2‐like ratio.

### 4.4 Obesity‐Induced Diabetic Model

Diabetes is an obesity related disease, hence, feeding mice with a diet enriched in fat is an established model to study metabolic disorders that resemble metabolic syndrome in humans [48].

We fed adult mice (8 to 12 weeks old) *ad‐libitum* with a fat‐enriched diet (HFD), where 60% of the calories are fat‐derived and 1.5% of cholesterol (D11103002i Research Diets) for 8 to 12 weeks. Mice were weighed every two weeks to evaluate weight gain.

### 4.5 In Vivo Metabolic Tests

Several metabolic parameters were analyzed in the Animal Facility at the CNIC, CNIO or the Animal Facility at Universidad de Santiago de Compostela. Glucose Tolerance Test (GTT) was performed as in [4] and allowed us to assess glucose metabolism status in animals. Mice were starved for 16 h, and basal glucose levels were measured by collecting 1 blood drop from the distal part of the tail and using a glucometer and glucose strips (Breeze2, Bayer). After basal measurement, we injected i.p 1 mg/g of glucose (Sigma) dissolved in PBS and measured glucose at 15, 30, 60, 90, and 120 min post‐injection. Glucose level values were expressed in mg/dl. Insulin tolerance test (ITT) was performed as in [4], this procedure allows us to know how peripheral tissues were responding to insulin and whether these tissues were sensitive or resistant to insulin. ITT is mechanically performed similarly to GTT, however, animals were starved for just 1 h, when basal glucose levels were quantified, and then 0.75 U/kg of insulin was injected. We used the same time points as GTT, 15, 30, 60, 90, and 120 min after insulin injection. Glucose levels are relativized to basal glucose levels and expressed as a percentage (%).

For insulin‐stimulated ^18^F‐FDG PET‐CT, fasted mice first received insulin followed by ^18^F‐FDG administration and imaging. PET images were quantified using the maximum standardized uptake value (SUV max), calculated as follows: SUV max = Maximum Voxel radioactivity Concentration (MBq/g)/Decay‐corrected injected Dose (Mbq)/Body Weight (g)

Tissue tracer uptake was normalized to the blood pool to reduce data variability, yielding the tissue‐to‐blood ratio (TBR). Tissue uptake was quantified in subcutaneous WAT, skeletal muscle, liver, heart, and BAT. Epididymal WAT was not quantified because of signal contamination from the adjacent urinary bladder. Interscapular BAT surface temperature was measured by infrared thermography under basal conditions.

For indirect calorimetry, mice were housed individually in metabolic cages after an acclimation period. Energy expenditure, respiratory exchange ratio/fatty‐acid oxidation, locomotor activity, and food intake were recorded across light and dark phases. Energy‐expenditure analyses were interpreted together with body‐composition data.

Magnetic Resonance Imaging (MRI) was performed by Dr. Rubén Nogueiras laboratory at Universidad de Santiago de Compostela. WT and *Mkk3* ^−/−^ mice were fed a HFD for 8 weeks and transferred to Santiago de Compostela, where mice acclimatized for 1 week before fat mass and lean mass were measured by MRI (Whole Body Composition Analyzer; EchoMRI, Houston, TX). Fat mass and lean mass values were expressed both in mass (g) and percentage (%) the latter relativized to full body weight.

### 4.6 Cell Culture

In this study, we isolated and cultured pre‐adipocytes and differentiated them to mature adipocytes.

Subcutaneous fat depots were surgically collected and submerged in Dulbecco´s Modified Eagle Medium (DMEM, Gibco). Adipose tissue was first mechanically disaggregated using tweezers and a razor and second enzymatically disaggregated using type‐A collagenase medium containing 2 mg/mL collagenase type‐A (Roche), 20 mg/mL BSA (Sigma), and 1X PBS (2 mL of solution per animal). Fat with collagenase A medium was incubated at 37°C in an end‐to‐end water bath. After disaggregation, the reaction was stopped by adding three volumes of DMEM supplemented with F12 (Gibco), 10% Fetal Bovine Serum (FBS) (Lonza), 2 mM L‐Glutamine (Sigma), and 100 U/mL penicillin/streptomycin (Sigma), cell suspension was passed through a 70 µm cell strainer (Falcon) to eliminate stroma and debris and centrifuged at 250*xg* for 8 min at RT. Pellet was collected, stored, and supernatant was re‐centrifuged at 400x*g* for 8 min at RT. Both pellets were combined, and cells were counted using a CasyTon cell counter.

Equal numbers of cells were seeded on 10 cm cell culture plates (Costar Corning) and placed in the incubator at 37°C with 5% of CO_2_.

After differentiation, adipocytes were starved for 22 h in DMEM containing 5% FBS and for an additional 2 h in serum‐free DMEM. Cells were treated as indicated with 0.5 mM palmitate and/or 10 ng/mL TNFα for 24 h. SB203580, rapamycin, and the selective p70S6K inhibitor PF‐4708671 were used at 10 µM under the conditions indicated in the figure legends. Cells were stimulated with 10 nM insulin for 10 min before lysis. For GLUT4 translocation, cells were stained for GLUT4 and Na^+^/K^+^‐ATPase, and using a Leica SP8 confocal microscope, the membrane‐to‐total GLUT4 signal was quantified by immunofluorescence and quantified using ImageJ software.

### 4.7 Histology

Liver, WAT, and BAT tissues samples were previously fixed in 10% formalin dilution for at least 48 h, samples were dehydrated in different concentrations of ethanol by the Histology Unit at CNIC and embedded in paraffin using a Tissue Embedding Center (Leica, EG1150). Paraffin blocks with tissue embedded were cut at 5 to 8 µm thickness using a microtome, and dried for 24 h at 37°C. Paraffin was removed and tissue hydrated again following sequential 2 min baths of xylol, 100% ethanol, 70% ethanol, 50% ethanol, and water. For hematoxylin/eosin staining, hematoxylin stains acidic structures such as cellular nuclei and eosin stains basic structures present in the cytoplasm [49]. Slides were hydrated, stained, dehydrated, and mounted using Permount medium (Fisher Scientific). Slides were observed using an optic microscope (Leica DM2500) at different magnifications, and pictures were taken using Leica Application Suite (L.A.S). For adipocyte area quantification ImageJ with the adipocount package was used.

### 4.8 Oil‐Red O Staining

Oil Red O (Sigma) is a lysochrome that stains neutral triglycerides and lipids in tissues [50]. Oil Red O was prepared at 0.7% dissolved in propylene glycol, filtered, and stored at 55°C. We used this stain in adipocyte culture previously fixed with 4% paraformaldehyde (diluted from 16% PFA, Sigma) for 2 h or OCT embedded liver or muscle tissues. Adipocytes were dehydrated following sequential baths of propylene glycol and stained with Oil Red O at RT for 2 h, shaking on a plate shaker. Excess Oil Red O was removed with water and observed with an optical microscope (Leica DM2500); pictures were taken using Leica Application Suite (L.A.S). For tissue staining, we make sections of 4% PFA fixed tissues embedded in OCT with a cryostat, remove OCT with PBS, incubate in propylene glycol for 10 min, stained with Oil Red O and used H&E as contrast staining.

Staining quantification was performed by adding equal volumes of methanol to each well, as methanol removes Oil Red O and acquires the red color. Color absorbance was measured in a spectrophotometer (BioRad Benchmark Plus) at 520 nm wavelength, and relativized to non‐differentiated WT adipocytes absorbance. Adipocyte plates were kept in water at 4°C for long term storage. Quantification of Oil Red O in tissues was performed with ImageJ.

### 4.9 Western Blot and Immunoprecipitation

Total proteins from white adipose tissue (WAT), liver and muscle were extracted in lysis buffer (50 mM Tris‐HCl pH 7.5, 1 mM EGTA, 1 mM EDTA pH 8.0, 50 mM sodium fluoride, 1 mM sodium glycerophosphate, 5 mM pyrophosphate, 0.27 M sucrose, 1% Triton X‐100, 0.1 mM PMSF, 0.1% β‐mercaptoethanol, 1 mM sodium ortovanadate, 1 µg/mL leupeptin, 1 µg/mL aprotinin). Tissues were homogenized with a Turrax (IKA T‐100), centrifuged at 14 000 rpm, 4°C for 20 min and the supernatant was collected.

Protein concentration from supernatants was quantified using the Bradford method [51]. This technique is based on a colorimetric reaction: Coomassie blue reagent (Coomassie G‐250, BioRad) turns blue when it interacts with proteins containing acidic and aromatic amino acids. This color shift can be measured by a spectrophotometer reading at 595 nm wavelength and extrapolated to a standard curve made with known concentrations of protein solution. 2 µL of our protein solution was added in a 96‐well plate (Corning Costar), and 200 µL of Coomassie reagent was used to develop color. The reaction was kept at room temperature (RT) for 5 min, and absorbance was detected on a spectrophotometer (BioRad Benchmark Plus).

Lysates were denatured in loading buffer (250 mM Tris‐HCl pH 6.8, 10% SDS, 30% glycerol, 0.01% bromophenol blue, 5% β‐mercaptoethanol) at 95°C for 5 min. Equal amounts of lysates were loaded in 10% to 12% polyacrylamide gels (30% Acrylamide/Bis Solution, 29:1, BioRad) and run at 150 V for 90 min. Gels were transferred to 0.2 µm pore nitrocellulose membrane (BioRad), blocked with 10% fat‐free milk powder for 45 min and probed overnight (ON) with the following primary antibodies at 1:1000 concentration: phospho AKT Thr308 (#9275), phospho AKT Ser473 (#9271), AKT (#9272), phospho p38 Thr180/Tyr182 (#9211), phospho S6K Thr389 (#9206), S6K (#2708), phospho S6 Ser240/244 (#5364), S6 (#2217), all from Cell Signaling Technologies, Vinculin (V4505) and p38α (SC‐535) from Sigma and Santa Cruz respectively. Anti‐rabbit or anti‐mouse conjugated with horseradish peroxidase secondary antibodies were used at 1:5000 concentration in BSA 5% and incubated 1 h at room temperature (RT). Western blot was developed using ECL detection reagent (Amersham ECL, GE Healthcare).

To improve specificity and signal strength, some proteins were immuno‐precipitated using G‐Sepharose beads. 20 µL of G‐Sepharose beads were incubated with 2 µg of IRS‐1 antibody. Beads and antibody were both incubated in a rotatory wheel at 4°C for 1 h. After incubation were centrifuged twice with 1X PBS at 14,000 rpm at RT on a benchtop centrifuge to wash samples, and one last time with lysis buffer. After washing beads, 750 µg of protein was added and incubated ON at 4°C in the rotatory wheel. The next day, samples were centrifuged, and the unbound fraction was collected and stored at –80°C. Precipitated beads were washed 3X with lysis buffer and denatured at 95°C for 5 min in loading buffer. We centrifuged samples, loaded supernatant onto pre‐cast 4%–10% acrylamide gels (Bio‐Rad), and transferred as described in SDS‐PAGE electrophoresis. Phospho‐IRS‐1 Ser307 (07‐247) and IRS‐1 (#2382) antibodies from Up‐state and Cell Signaling Technologies, respectively, were used.

### 4.10 RNA Isolation and qPCR

mRNA from tissues and cells was extracted following instructions from the kit manufacturer (Qiagen RNA Mini Extraction Kit). QIAzol lysis reagent and Turrax (IKA T‐100) were used on tissues and cells to homogenize samples. mRNA was quantified using a NanoDrop spectrophotometer (ND‐1000, Thermo Scientific). For retrotranscription, 100 ng of mRNA either from WAT or adipocytes was reverse transcribed to cDNA using the High‐Capacity cDNA Reverse Transcription Kit (Thermo Fisher) following manufacturer instructions. The reaction was set to 10 min at 25°C, 2 h at 37°C and 5 min at 85°C. qPCR was performed using specific primers and the Fast SYBR Green system (Applied Biosystems) or TaqMan Assays (Applied Biosystems) on a 7900HT Fast Real‐time PCR thermal cycler (Applied Biosystems). A dissociation curve cycle was used after each reaction to verify primer specificity and purity. Expression levels were normalized to S18 and actin, and mRNA expression was calculated by interpolating Ct values on a standard curve of mRNA.

### 4.11 Luminex Circulating Hormone and Adipokine Analysis

We analyzed levels of insulin, C‐peptide, adiponectin, and leptin, two circulating cytokines and two adipokines respectively, from serum extracted from blood obtained from control and bariatric surgery patients.

Briefly, Luminex is based on microspheres coated with specific antibodies, these microspheres are internally dyed with a combination of red and infrared dyes. Different combinations of red and infrared dyes allow for the detection of each microsphere specifically, allowing the detection of multiple analytes in the same sample.

Serum cytokine and adipokine concentrations were measured by multiplexed Enzyme‐Linked Immunosorbent Assay (ELISA) using a Luminex 200 IS analyzer (Millipore) following manufacturer´s instructions.

### 4.12 Statistical Analysis

Data are presented as mean ± SEM unless otherwise indicated. Statistical analyses were performed using GraphPad Prism and SPSS. Sample size, the definition of biological replicates and the statistical test used for each experiment are provided in the corresponding figure legend.

Two‐group comparisons were performed using two‐sided Student's *t*‐tests, with Welch's correction when variances were unequal. Experiments with more than two groups or repeated measurements were analysed by one‐ or two‐way ANOVA followed by the indicated multiple‐comparison procedure. Human associations were evaluated using Spearman or Pearson correlation as appropriate. For multivariable analysis of HbA1c, MKK3 expression was log2‐transformed and linear regression models were adjusted for age, sex, BMI and type 2 diabetes status; sensitivity models replaced diabetes status with antidiabetic treatment and additionally considered histological steatosis when sample availability permitted. *p* < 0.05 was considered statistically significant.

## Conflicts of Interest

The authors declare no conflicts of interest.

## Supporting information




**Supporting File 1**: advs77744‐sup‐0001‐SuppMat.docx.

**Supporting File 2**: advs77744‐sup‐0002‐TableS1.xlsx.

## Data Availability

The data that support the findings of this study are available on request from the corresponding author. The data are not publicly available due to privacy or ethical restrictions.

## References

[1] R. Divella , R. De Luca , I. Abbate , E. Naglieri , and A. Daniele , “Obesity and Cancer: The Role of Adipose Tissue and Adipo‐Cytokines‐Induced Chronic Inflammation,” Journal of Cancer 7, no. 15 (2016): 2346–2359, 10.7150/jca.16884. PMC5166547 27994674

[2] B. B. Kahn and J. S. Flier , “Obesity and Insulin Resistance,” Journal of Clinical Investigation 106, no. 4 (2000): 473–481, 10.1172/JCI10842. PMC380258 10953022

[3] G. van Hall , A. Steensberg , M. Sacchetti , et al., “Interleukin‐6 Stimulates Lipolysis and Fat Oxidation in Humans,” Journal of Clinical Endocrinology and Metabolism 88 (2003): 3005–3010. 10.1210/jc.2002-021687 12843134

[4] G. Sabio , M. Das , A. Mora , et al., “A Stress Signaling Pathway in Adipose Tissue Regulates Hepatic Insulin Resistance,” Science 322, no. 5907 (2008): 1539–1543, 10.1126/science.1160794. PMC2643026 19056984

[5] E. Manieri and G. Sabio , “Stress Kinases in the Modulation of Metabolism and Energy Balance,” Journal of Molecular Endocrinology 55, no. 2 (2015): R11–R22, 10.1530/JME-15-0146. PMC5176079 26363062

[6] C. J. Carlson and C. M. Rondinone , “Pharmacological Inhibition of p38 MAP Kinase Results in Improved Glucose Uptake in Insulin‐resistant 3T3‐L1 Adipocytes,” Metabolism 54, no. 7 (2005): 895–901, 10.1016/j.metabol.2005.02.003. 15988698

[7] N. Matesanz , I. Nikolic , M. Leiva , et al., “p38α Blocks Brown Adipose Tissue Thermogenesis Through p38δ Inhibition,” PLoS Biology 16, no. 7 (2018): 2004455, 10.1371/journal.pbio.2004455. PMC6051667 29979672

[8] C. de Alvaro , T. Teruel , R. Hernandez , and M. Lorenzo , “Tumor Necrosis Factor Alpha Produces Insulin Resistance in Skeletal Muscle by Activation of Inhibitor kappaB Kinase in a p38 MAPK‐dependent Manner,” Journal of Biological Chemistry 279 (2004): 17070–17078. 10.1074/jbc.M312021200 14764603

[9] G. Li , E. J. Barrett , M. O. Barrett , W. Cao , and Z. Liu , “Tumor Necrosis Factor‐α Induces Insulin Resistance in Endothelial Cells via a p38 Mitogen‐Activated Protein Kinase‐Dependent Pathway,” Endocrinology 148, no. 7 (2007): 3356–3363, 10.1210/en.2006-1441. 17446186

[10] X. Zhang , L. Fan , J. Wu , et al., “Macrophage p38α Promotes Nutritional Steatohepatitis Through M1 Polarization,” Journal of Hepatology 71, no. 1 (2019): 163–174, 10.1016/j.jhep.2019.03.014. 30914267

[11] B. González‐Terán , N. Matesanz , I. Nikolic , et al., “p38γ and p38δ Reprogram Liver Metabolism by Modulating Neutrophil Infiltration,” The EMBO Journal 35, no. 5 (2016): 536–552, 10.15252/embj.201591857. PMC4772851 26843485

[12] M. Crespo , I. Nikolic , A. Mora , et al., “Myeloid p38 Activation Maintains Macrophage–Liver Crosstalk and BAT Thermogenesis Through IL‐12–FGF21 Axis,” Hepatology 77, no. 3 (2023): 874–887, 10.1002/hep.32581. PMC9936978 35592906

[13] M. Crespo , B. Gonzalez‐Teran , and I. Nikolic , “Neutrophil Infiltration Regulates Clock‐gene Expression to Organize Daily Hepatic Metabolism,” (2020): 59258. 10.7554/eLife.59258 PMC7723411 33287957

[14] L. Herrera‐Melle , B. Cicuéndez , J. A. López , et al., “p38α Kinase Governs Muscle Strength Through PGC1α in Mice,” Acta Physiologica 240, no. 11 (2024): 14234, 10.1111/apha.14234. 39361268

[15] C. Folgueira , L. Herrera‐Melle , J. A. López , et al., “Remodeling p38 Signaling in Muscle Controls Locomotor Activity via IL‐15,” Science Advances 10, no. 33 (2024): adn5993, 10.1126/sciadv.adn5993. PMC11323882 39141732

[16] N. Matesanz , E. Bernardo , R. Acín‐Pérez , et al., “MKK6 Controls T3‐Mediated Browning of White Adipose Tissue,” Nature Communications 8, no. 1 (2017): 856, 10.1038/s41467-017-00948-z. PMC5636784 29021624

[17] I. Nikolic , I. Ruiz‐Garrido , M. Crespo , et al., “Lack of p38 Activation in T Cells Increases IL‐35 and Protects Against Obesity by Promoting Thermogenesis,” EMBO Reports 25, no. 6 (2024): 11, 10.1038/s44319-024-00149-y. PMC11169359 38730210

[18] M. Aouadi , K. Laurent , M. Prot , Y. Le Marchand‐Brustel , B. Binétruy , and F. Bost , “Inhibition of p38MAPK Increases Adipogenesis From Embryonic to Adult Stages,” Diabetes 55, no. 2 (2006): 281–289, 10.2337/diabetes.55.02.06.db05-0963. 16443758

[19] S. Turban , V. A. Beardmore , J. M. Carr , et al., “Insulin‐stimulated Glucose Uptake Does Not Require p38 Mitogen‐activated Protein Kinase in Adipose Tissue or Skeletal Muscle,” Diabetes 54, no. 11 (2005): 3161–3168, 10.2337/diabetes.54.11.3161. 16249440

[20] Y. Kuma , G. Sabio , J. Bain , N. Shpiro , R. Márquez , and A. Cuenda , “BIRB796 inhibits all p38 MAPK Isoforms in Vitro and in Vivo,” Journal of Biological Chemistry 280, no. 20 (2005): 19472–19479, 10.1074/jbc.M414221200. 15755732

[21] R. Romero‐Becerra , A. Mora , E. Manieri , et al., “MKK6 deficiency Promotes Cardiac Dysfunction Through MKK3‐p38gamma/Delta‐mTOR Hyperactivation,” Elife 11 (2022): 75250. 10.7554/eLife.75250 PMC9381040 35971771

[22] G. Remy , A. M. Risco , F. A. Iñesta‐Vaquera , et al., “Differential Activation of p38MAPK Isoforms by MKK6 and MKK3,” Cellular Signalling 22, no. 4 (2010): 660–667, 10.1016/j.cellsig.2009.11.020. 20004242

[23] R. Romero‐Becerra , F. M. Cruz , A. Mora , et al., “p38γ/δ Activation Alters Cardiac Electrical Activity and Predisposes to Ventricular Arrhythmia,” Nature Cardiovascular Research 2, no. 12 (2023): 1204–1220, 10.1038/s44161-023-00368-x. 39196141

[24] S. C. Ruffolo , P. K. A. Forsell , X. Yuan , et al., “Basal Activation of p70S6K Results in Adipose‐specific Insulin Resistance in Protein‐tyrosine Phosphatase 1B–/– Mice,” Journal of Biological Chemistry 282, no. 42 (2007): 30423–30433, 10.1074/jbc.M700697200. 17664276

[25] I. Nikolic , M. Leiva , and G. Sabio , “The Role of Stress Kinases in Metabolic Disease,” Nature Reviews Endocrinology 16, no. 12 (2020): 697–716, 10.1038/s41574-020-00418-5. 33067545

[26] S. Cinti , G. Mitchell , G. Barbatelli , et al., “Adipocyte Death Defines Macrophage Localization and Function in Adipose Tissue of Obese Mice and Humans,” Journal of lipid research 46, no. 11 (2005): 2347–2355, 10.1194/jlr.M500294-JLR200. 16150820

[27] L. Boutens , G. J. Hooiveld , S. Dhingra , R. A. Cramer , M. G. Netea , and R. Stienstra , “Unique Metabolic Activation of Adipose Tissue Macrophages in Obesity Promotes Inflammatory Responses,” Diabetologia 61, no. 4 (2018): 942–953, 10.1007/s00125-017-4526-6. PMC6448980 29333574

[28] V. Aguirre , E. D. Werner , J. Giraud , Y. H. Lee , S. E. Shoelson , and M. F. White , “Phosphorylation of Ser307 in Insulin Receptor Substrate‐1 Blocks Interactions With the Insulin Receptor and Inhibits Insulin Action,” Journal of Biological Chemistry 277, no. 2 (2002): 1531–1537, 10.1074/jbc.M101521200. 11606564

[29] L. R. Pearce , G. R. Alton , D. T. Richter , et al., “Characterization of PF‐4708671, a Novel and Highly Specific Inhibitor of p70 Ribosomal S6 Kinase (S6K1),” Biochemical Journal 431, no. 2 (2010): 245–255, 10.1042/BJ20101024. 20704563

[30] O. J. Shah , Z. Wang , and T. Hunter , “Inappropriate Activation of the TSC/Rheb/mTOR/S6K Cassette Induces IRS1/2 Depletion, Insulin Resistance, and Cell Survival Deficiencies,” Current Biology 14, no. 18 (2004): 1650–1656, 10.1016/j.cub.2004.08.026. 15380067

[31] L. S. Harrington , G. M. Findlay , A. Gray , et al., “The TSC1‐2 Tumor Suppressor Controls Insulin–PI3K Signaling via Regulation of IRS Proteins,” The Journal of Cell Biology 166, no. 2 (2004): 213–223, 10.1083/jcb.200403069. PMC2172316 15249583

[32] S. H. Um , F. Frigerio , M. Watanabe , et al., “Absence of S6K1 Protects Against Age‐ and Diet‐induced Obesity While Enhancing Insulin Sensitivity,” Nature 431, no. 7005 (2004): 200–205, 10.1038/nature02866. 15306821

[33] M. Zheng , Y.‐H. Wang , X.‐N. Wu , et al., “Inactivation of Rheb by PRAK‐mediated Phosphorylation Is Essential for Energy‐Depletion‐Induced Suppression of mTORC1,” Nature Cell Biology 13, no. 3 (2011): 263–272, 10.1038/ncb2168. PMC3070924 21336308

[34] B. González‐Terán , J. A. López , E. Rodríguez , et al., “p38γ and δ Promote Heart Hypertrophy by Targeting the mTOR‐Inhibitory Protein DEPTOR for Degradation,” Nature Communications 7, no. 1 (2016): 10477, 10.1038/ncomms10477. PMC5476828 26795633

[35] X.‐N. Wu , X.‐K. Wang , S.‐Q. Wu , et al., “Phosphorylation of Raptor by p38β Participates in Arsenite‐induced Mammalian Target of Rapamycin Complex 1 (mTORC1) Activation,” Journal of Biological Chemistry 286, no. 36 (2011): 31501–31511, 10.1074/jbc.M111.233122. PMC3173133 21757713

[36] G. Sumara , I. Formentini , S. Collins , et al., “Regulation of PKD by the MAPK p38δ in Insulin Secretion and Glucose Homeostasis,” Cell 136, no. 2 (2009): 235–248, 10.1016/j.cell.2008.11.018. PMC2638021 19135240

[37] A. Ittner , H. Block , C. A. Reichel , et al., “Regulation of PTEN Activity by p38δ‐PKD1 Signaling in Neutrophils Confers Inflammatory Responses in the Lung,” Journal of Experimental Medicine 209, no. 12 (2012): 2229–2246, 10.1084/jem.20120677. PMC3501357 23129748

[38] H.‐T. Lu , D. D. Yang , M. Wysk , et al., “Defective IL‐12 Production in Mitogen‐Activated Protein (MAP) Kinase Kinase 3 (Mkk3)‐Deficient Mice,” The EMBO Journal 18, no. 7 (1999): 1845–1857, 10.1093/emboj/18.7.1845. PMC1171270 10202148

[39] J. A. Nick , “Selective Activation and Functional Significance of p38α Mitogen‐Activated Protein Kinase in Lipopolysaccharide‐Stimulated Neutrophils,” Journal of Clinical Investigation 103, no. 6 (1999): 851–858, 10.1172/JCI5257. PMC408145 10079106

[40] A. Srivastava , A. S. Shinn , P. J. Lee , and P. Mannam , “MKK3 mediates Inflammatory Response Through Modulation of Mitochondrial Function,” Free Radical Biology and Medicine 83 (2015): 139–148, 10.1016/j.freeradbiomed.2015.01.035. PMC4441852 25697779

[41] B. González‐Terán , J. R. Cortés , E. Manieri , et al., “Eukaryotic Elongation Factor 2 Controls TNF‐α Translation in LPS‐Induced Hepatitis,” Journal of Clinical Investigation 123, no. 1 (2013): 164–178, 10.1172/JCI65124. PMC3533299 23202732

[42] C. Cabane , W. Englaro , K. Yeow , M. Ragno , and B. Dérijard , “Regulation of C2C12 Myogenic Terminal Differentiation by MKK3/p38α Pathway,” American Journal of Physiology‐Cell Physiology 284, no. 3 (2003): C658–C666, 10.1152/ajpcell.00078.2002. 12444016

[43] C. M. Schonhoff , S. W. Park , C. R. L. Webster , and M. S. Anwer , “p38 MAPK α and β Isoforms Differentially Regulate Plasma Membrane Localization of MRP2,” American Journal of Physiology‐Gastrointestinal and Liver Physiology 310, no. 11 (2016): G999–G1005, 10.1152/ajpgi.00005.2016. PMC4935486 27012769

[44] L. Bian , “MAP2K3 is Associated With Body Mass Index in American Indians and Caucasians and May Mediate Hypothalamic Inflammation,” Human Molecular Genetics 22, no. 21 (2013): 4438–4449, 10.1093/hmg/ddt291. PMC3792696 23825110

[45] W. He , Y. Barak , A. Hevener , et al., “Adipose‐Specific Peroxisome Proliferator‐Activated Receptor γ Knockout Causes Insulin Resistance in Fat and Liver but not in Muscle,” Proceedings of the National Academy of Sciences 100, no. 26 (2003): 15712–15717, 10.1073/pnas.2536828100. PMC307633 14660788

[46] J. Eguchi , X. Wang , S. Yu , et al., “Transcriptional Control of Adipose Lipid Handling by IRF4,” Cell Metabolism 13, no. 3 (2011): 249–259, 10.1016/j.cmet.2011.02.005. PMC3063358 21356515

[47] B. E. Clausen , C. Burkhardt , W. Reith , R. Renkawitz , and I. Forster , “Conditional Gene Targeting in Macrophages and Granulocytes Using LysMcre Mice,” Transgenic Research 8, no. 4 (1999): 265–277, 10.1023/A:1008942828960. 10621974

[48] R. Buettner , J. Scholmerich , and L. C. Bollheimer , “High‐fat Diets: Modeling the Metabolic Disorders of Human Obesity in Rodents,” obesity 15, no. 4 (2007): 798–808, 10.1038/oby.2007.608. 17426312

[49] A. H. Fischer , K. A. Jacobson , J. Rose , and R. Zeller , “Hematoxylin and Eosin Staining of Tissue and Cell Sections,” Cold Spring Harbor Protocols 2008 (2008): pdbprot4986. 10.1101/pdb.prot4986 21356829

[50] G. J. Hausman , “Techniques for Studying Adipocytes,” Stain technology 56, no. 3 (1981): 149–154, 10.3109/10520298109067302. 6168034

[51] M. M. Bradford , “A Rapid and Sensitive Method for the Quantitation of Microgram Quantities of Protein Utilizing the Principle of Protein‐Dye Binding,” Analytical biochemistry 72, no. 1‐2 (1976): 248–254, 10.1016/0003-2697(76)90527-3. 942051

