# Supplementary material for: An Adipocyte MKK3–p38β Axis Preserves Insulin Responsiveness in Mice and Is Reciprocally Altered in Human Obesity
Source: Adv Sci (Weinh). 2026 Oct 28:e20388. Online ahead of print. doi: 10.1002/advs.202520388 (PMC13717117; doi:10.1002/advs.202520388)
Supplement: Supplementary file 1 [file ADVS-9999-e20388-s001.docx]

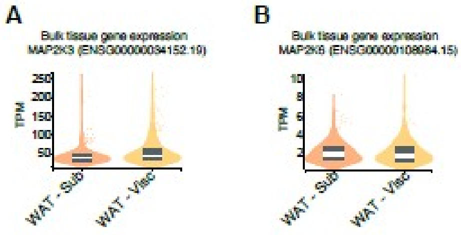

C

MKK3-MKK6 correlation

	MKK3	MKK6	Age (years)	Weight (kg)	BMI	Glucose (mg/dl)	Total chol. (mg/dl)	TG (mg/dl)	HDL (mg/dl)	LDL (mg/dl)
MKK3 Rho	1	.265*	-0.144	.265*	.304*	0	0.094	-0.030	0.049	-0.05
P	.	0.037	0.265	0.037	0.016	0.998	0.501	0.896	0.728	0.726
N	62	62	62	62	62	61	54	54	53	51
MKK6 Rho	.265*	1	-0.115	-0.035	0.062	0.046	0.06	0.101	-0.266	0.064
P	0.037	.	0.373	0.906	0.63	0.733	0.668	0.467	0.054	0.656
N	62	62	62	62	62	61	54	54	53	51

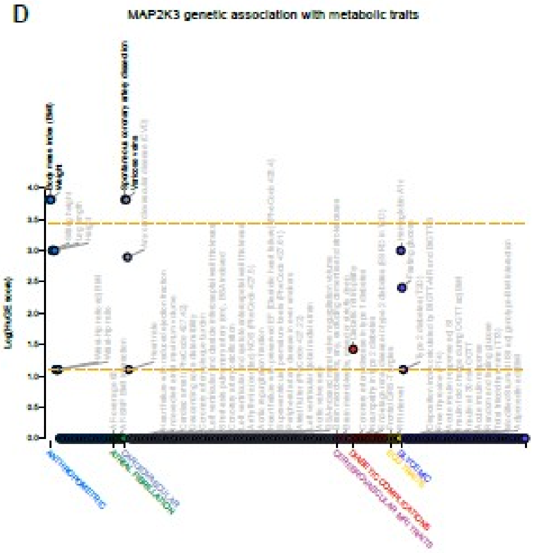

Supplementary Figure 1.
Human adipose expression and genetic associations of MAP2K3/MKK3 and MAP2K6/MKK6. A, B. MAP2K3/MKK3 (A) and MAP2K6/MKK6 (B) mRNA expression in human subcutaneous and visceral adipose tissue from GTEx. C. Correlation analysis of MKK3 and MKK6 expression with anthropometric and metabolic parameters in human visceral adipose tissue. D. Genetic associations of MAP2K3 with metabolic traits, including BMI, body weight, fasting glucose and HbA1c. Data were obtained from the Common Metabolic Diseases Knowledge Portal (CMDKP).

Supplementary Figure 2

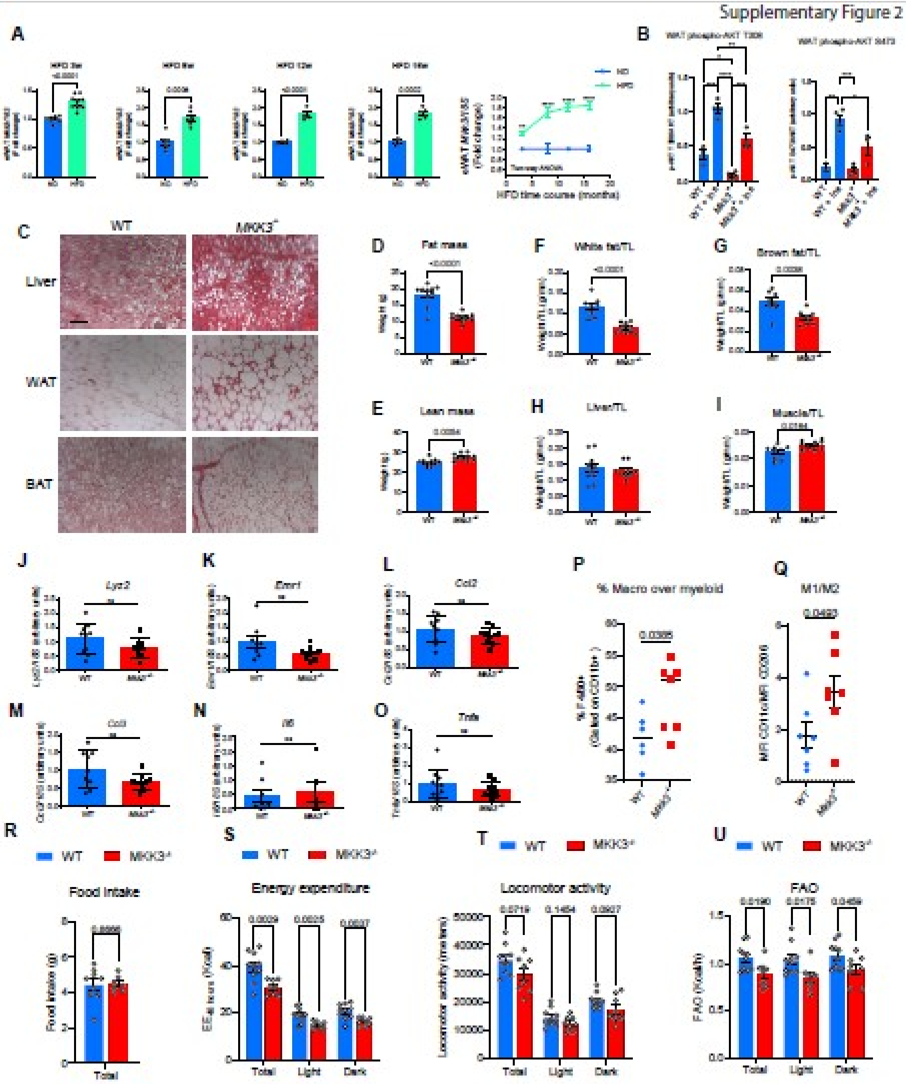

Supplementary Figure 2.
Metabolic and adipose-tissue phenotype of germline Mkk3$^{-/-}$ mice. **A.** Time course of Mkk3 expression in eWAT from WT mice fed ND or HFD for 3, 8, 12 or 16 weeks. Right panel shows fold change over time. Data are presented as mean ± SEM, n = 3–9. **B.** Quantification of Akt Thr308 and Ser473 phosphorylation in WAT immunoblots shown in Fig. 2H. C. Representative H&E images of liver, WAT and BAT from HFD-fed WT and Mkk3$^{-/-}$ mice. Scale bar, 100 µm. **D, E.** Fat mass (D) and lean mass (E) measured by MRI. **F–I.** WAT (F), BAT (G), liver (H) and skeletal muscle (I) weights normalized to tibial length. **J–O.** Expression of Lyz2 (J), Emr1 (K), Ccl2 (L), Ccl3 (M), Il6 (N) and Tnfα (O) in WAT. **P, Q.** Flow-cytometry analysis of macrophage abundance (P) and M1-like/M2-like macrophage ratio (Q) in the eWAT stromal vascular fraction. **R–U.** Food intake (R), energy expenditure (S), locomotor activity (T) and fatty-acid oxidation (U) measured by indirect calorimetry. Data are presented as mean ± SEM; statistical tests and n values are indicated in the corresponding panels.

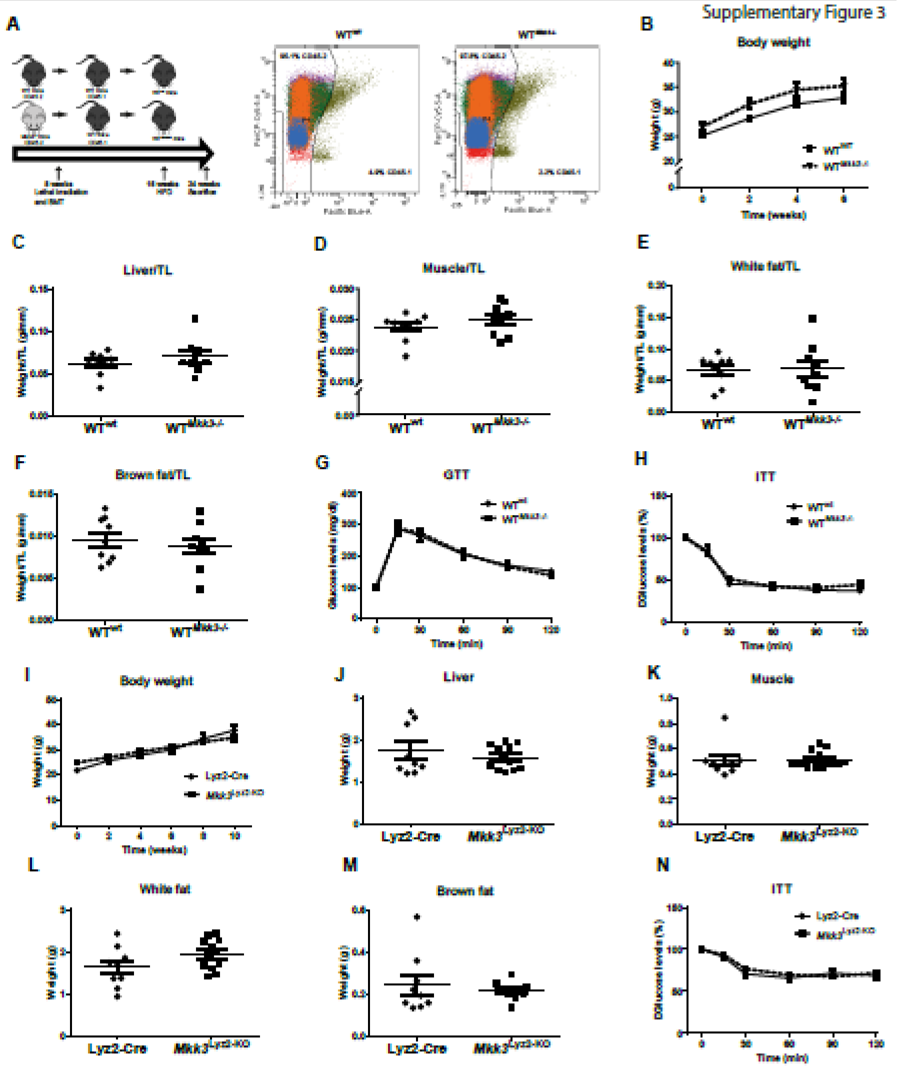

Supplementary Figure 3.
A. Schematic of the experimental approach (left) and cytometry analysis (right plot) of acceptor stromal vascular fraction, plots showing more than 95% efficiency of WT or *Mkk3*$^{-/-}$ bone marrow reconstitution transplanted into 8 month old lethally irradiated WT recipient congenic mice. **B.** Weight gain after 6 weeks of HFD of WT^{WT} and WT$^{Mkk3-/-}$ mice two months after bone marrow transplantation. Data are presented as mean ± SEM (n = 9), Two-way ANOVA coupled with Bonferroni post-test. **C-F.** Unaltered liver (C), muscle (D), white (E), and brown (F) adipose tissue weight differences relative to tibial length in WT$^{Mkk3-/-}$ mice after six weeks of HFD feeding. Data are presented as mean ± SEM, n=9, Student´s t-test coupled with the Bonferroni post-test. **G, H.** GTT and ITT of WT$^{Mkk3-/-}$ mice fed an HFD for 6 weeks showed no differences in glucose or insulin tolerance. Data are presented as direct measure of glucose in GTT and decrease vs. basal glycemia expressed as percentage in ITT, mean ± SEM, n=9, Two-way ANOVA coupled to Bonferroni post-test. **I.** Unchanged weight gain of myeloid-specific *Mkk3* conditional mice (*Mkk3*$^{Lyz2-KO}$) fed an HFD for 10 weeks. Data are presented as the mean ± SEM, n=9-12, Two-way ANOVA coupled with Bonferroni post-test. **J-M.** No differences in liver (J), muscle (K), white (L), and brown (M) adipose tissue weights were observed in *Mkk3*$^{Lyz2-KO}$ mice. **N.** The insulin tolerance test showed no differences in the insulin sensitivity of *Mkk3*$^{Lyz2-KO}$ mice fed an HFD for 12 weeks. Data are presented as the mean ± SEM, n=9-12, Two-way ANOVA coupled with Bonferroni post-test.

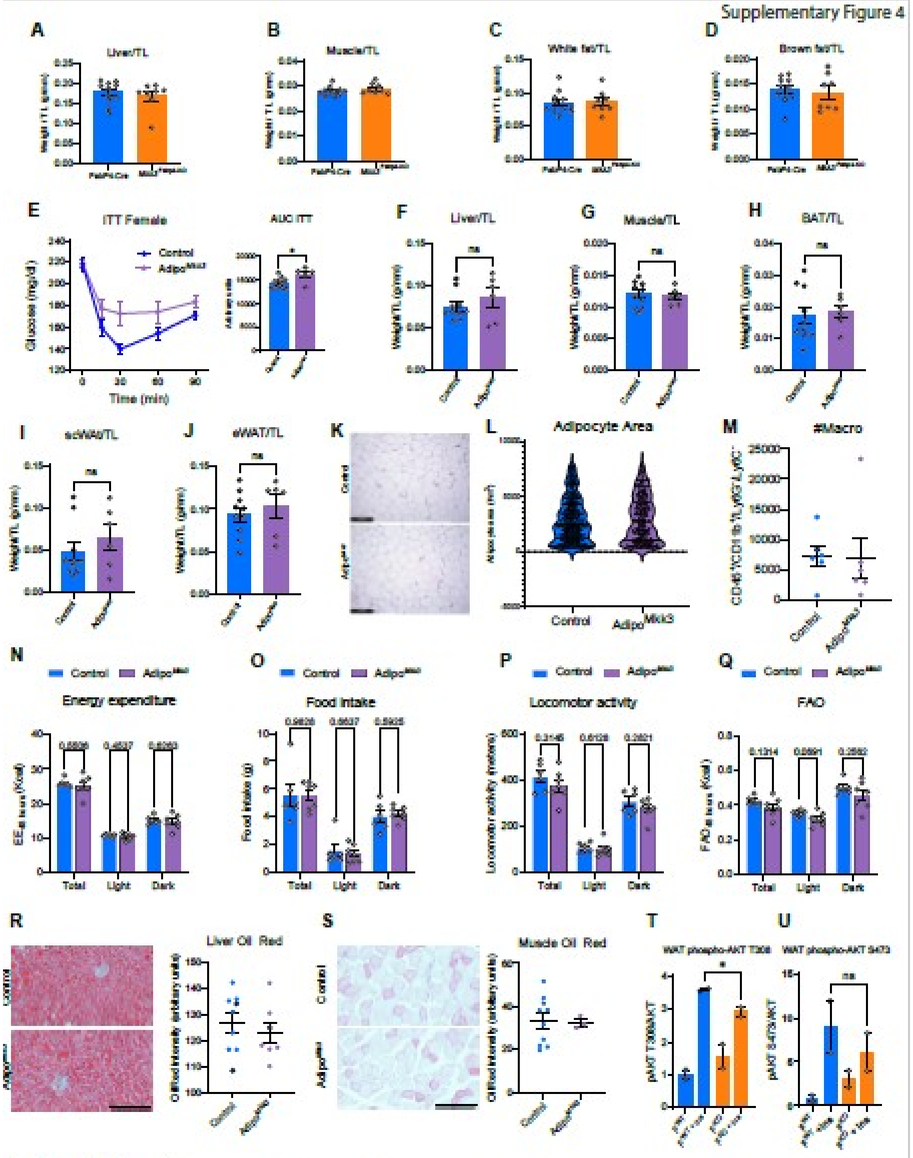

Supplementary Figure 4.
Additional characterization of adipocyte-specific Mkk3-deficient mice. A-D. Liver (A), skeletal muscle (B), WAT (C), and BAT (D) weights normalized to tibial lenght in HFD-fed Fabp4-Cre control and Mkk3^{Fabp4KO} mice. E. ITT and corresponding AUC in female control and Adipo^{Mkk3} Female HFD-fed for 25w Adipo^{Mkk3} mice after prolonged HFD feeding. Data are presented as mean ± SEM, n = 5-11. F-J. Liver (F), skeletal muscle (G), BAT (H), scWAT (I) and eWAT (J) weights normalized to tibial lenght in HFD-fed control and Adipo^{Mkk3} mice. K, L. Representative H&E staining of eWAT (K) and adipocyte-area quantification (L). M. Flow cytometry quantification of macrophage abundance in eWAT stromal vascular fraction (n = 6). N-Q. Energy expenditure (N), food intake (O), locomotor activity (P) and fatty-acid oxidation (Q) measured by indirect calorimetry. R, S. Representative Oil Red O staining and quantification in liver (R) and skeletal muscle (S). T, U. Quantification of Akt Thr308 (T) and Ser473 (U) phosphorylation in WAT immunoblots shown in Fig. 3G. Data are presented as mean ± SEM; *P < 0.05.

Supplementary Figure 5

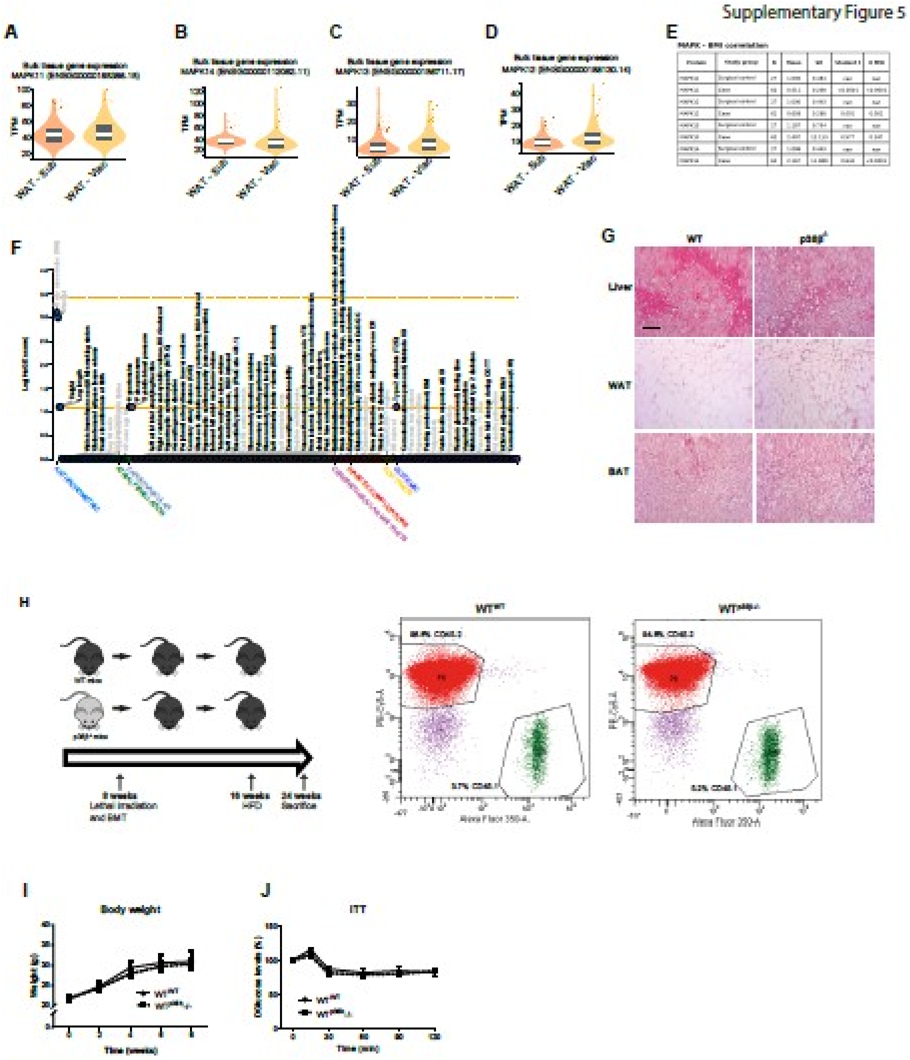

Supplementary Figure 5

Expression, genetic associations and metabolic phenotype of p38β. A–D. MAPK11/p38β (A), MAPK14/p38α (B), MAPK12/p38γ (C) and MAPK13/p38δ (D) mRNA expression in human subcutaneous and visceral adipose tissue from GTEx. E. Correlation analysis of MAPK11, MAPK14, MAPK12 and MAPK13 expression with BMI in human visceral adipose tissue. F. Genetic associations of MAPK11 with metabolic traits, including BMI, body weight and type 2 diabetes. Data were obtained from the Common Metabolic Diseases Knowledge Portal (CMDKP). G. Representative H&E images of liver, WAT and BAT from HFD-fed WT and p38β-/- mice; increased leukocyte infiltration is observed in WAT from p38β-/- mice. Scale bar, 100 μm. H. Schematic of the bone-marrow transplantation protocol and flow-cytometric analysis showing donor-derived reconstitution in the stromal vascular fraction of recipient WAT. I. Body weight of WT recipients reconstituted with WT or p38β-/- bone marrow during HFD feeding. J. ITT showing comparable insulin responsiveness in mice reconstituted with WT or p38β-/- bone marrow. Data are presented as mean ± SEM, n = 8–9; two-way ANOVA followed by Bonferroni's post hoc test.
